# Detection of a four-carbon sugar in interstellar space

**DOI:** 10.1038/s41550-026-02905-7

**Published:** 2026-07-13

**Authors:** Izaskun Jiménez-Serra, Juan García de la Concepción, Herma M. Cuppen, Marta Rey-Montejo, Miguel Sanz-Novo, Víctor M. Rivilla, Jesús Martín-Pintado, Andrés Megías, Carlos Briones, David San Andrés, Laura Colzi, Shaoshan Zeng, Sergio Martín, Joseph Salaris, Antonio Martínez-Henares, Álvaro López-Gallifa, Miguel Angel Requena-Torres, Belén Tercero, Pablo de Vicente, Aran Insausti, Elena R. Alonso, Emilio J. Cocinero

**Affiliations:** 1 Center for Astrobiology (CAB), CSIC-INTA, Torrejón de Ardoz, Spain; 2 Departamento de Química Orgánica e Inorgánica, Facultad de Ciencias and IACYS-Green Chemistry and Sustainable Development Unit, University of Extremadura, Badajoz, Spain; 3 Institute for Molecules and Materials, Radboud University, Nijmegen, the Netherlands; 4 Departamento de Física de la Tierra y Astrofísica, Facultad de Ciencias Físicas, Universidad Complutense de Madrid, Madrid, Spain; 5 Center for Astrochemical Studies, Max-Planck-Institut für extraterrestrische Physik, Garching bei Munchen, Germany; 6 Star and Planet Formation Laboratory, Cluster for Pioneering Research, Wako, Japan; 7 European Southern Observatory, Santiago, Chile; 8 Joint ALMA Observatory, Santiago, Chile; 9 Department of Physics, Astronomy and Geosciences, Towson University, Towson, MD USA; 10 Observatorio Astronómico Nacional (OAN-IGN), Madrid, Spain; 11 Observatorio de Yebes (OY-IGN), Yebes, Spain; 12 Department of Physical Chemistry, Faculty of Science and Technology, University of the Basque Country (EHU), Leioa, Spain; 13 Biofisika Institute, CSIC, EHU, Leioa, Spain; 14 Grupo de Espectroscopía Molecular, Edificio Quifima, Laboratorios de Espectroscopía y Bioespectroscopía, Universidad de Valladolid, Valladolid, Spain

**Keywords:** Interstellar medium, Astrobiology

## Abstract

Sugars are essential biomolecules, serving as metabolic fuels, nucleic acid backbone components and structural or energy-storage polymers. A central question in origin-of-life research is how monosaccharides formed on the primitive Earth, as laboratory experiments under prebiotic conditions yield insufficient concentrations. The detection of ribose, glucose and other monosaccharides in asteroids and meteorites suggests an exogenous origin, possibly in the interstellar medium (ISM) before meteoritic parent-body formation. However, no sugar has been observed in the ISM so far. Here we report the discovery of erythrulose, a chiral four-carbon ketose, in the ISM. The detection was achieved through ultrasensitive, broadband spectral surveys of the Galactic Centre molecular cloud G+0.693−0.027, using the Yebes 40 m and IRAM 30 m telescopes. Erythrulose appears to be at least eight times more abundant than analogous three-carbon sugars, which remain undetected in our ultrasensitive observations. Quantum chemical and astrochemical models indicate that erythrulose forms efficiently on interstellar dust grains from simpler two-carbon aldehydes and alcohols. As ketoses readily isomerize into aldoses in aqueous conditions, interstellar erythrulose could have contributed to the sugar inventory available for early metabolic and replication processes.

## Abstract (web summary)

A monosaccharide sugar has been detected in an interstellar cloud: the chiral ketose erythrulose. Observations and chemical modelling show that it forms efficiently on dust grains, supporting an interstellar origin for prebiotic sugars linked to early life.

## Main

The interstellar medium (ISM) is an impressive chemical factory in which more than 340 molecules have been detected so far^1^, including large aromatic species^2–4^. Several of these molecules, such as urea, hydroxylamine and ethanolamine^5–9^, are directly connected to origin-of-life chemistry because they are considered precursors of ribonucleosides and lipids^10–13^. Sugars are also central molecules to prebiotic chemistry^11,14,15^; however, they are usually introduced ad hoc (that is, as inputs) in prebiotic reaction schemes because their formation under plausible early-Earth conditions remains inefficient ^16–19^.

The detection of bio-essential sugars such as ribose and glucose in primitive meteorites and in asteroid Bennu suggests that at least part of the sugar inventory available on the early Earth may have had an exogenous origin^20,21^. A natural possibility is that these sugars, or their precursors, formed before parent-body accretion. However, no sugar has so far been reported in the ISM. Glycolaldehyde (HOCH_2_CHO) is widespread in interstellar space^22–24^ and has often been discussed in this context because of its structural relationship to aldose sugars. Yet, it is a hydroxyaldehyde rather than a true saccharide^25^.

A major obstacle to searching for interstellar sugars has been the lack of accurate gas-phase rotational data. Such data are essential for astronomical identification, but obtaining them was long considered extremely challenging because sugars are thermally fragile and strongly hygroscopic, which hampers their manipulation and conventional thermal vaporization. This limitation has recently been overcome using ultrafast laser vaporization, which enabled the gas-phase rotational characterization of ribose, 2-deoxyribose and erythrulose^26–28^. These laboratory data opened the way to sensitive astronomical searches for sugars with three and four carbon atoms (that is, C3 and C4 sugars)—for example, glyceraldehyde (HOCH_2_CH(OH)CHO), dihydroxyacetone (HOCH_2_COCH_2_OH) and erythrulose (HOCH_2_CH(OH)COCH_2_OH)—although previous searches remained unsuccessful^6,28–31^.

Here, we report the discovery of erythrulose—the only C4 ketose—in the ISM, towards the molecular cloud G+0.693−0.027 (hereafter G+0.693), located in the Galactic Centre region at a distance of ~8.2 kpc from us^32^. The identification was enabled by an ultrasensitive broadband spectral survey with the Yebes 40 m and the IRAM 30 m radiotelescopes covering more than 91 GHz across the 7 mm, 3 mm and 2 mm atmospheric windows (Methods). G+0.693 is one of the richest molecular reservoirs known in the Galaxy^6,33,34^ and has yielded numerous detections of new interstellar species of prebiotic interest in recent years^8,35,36^.

## Results

### Detection of erythrulose in G+0.693

**Fig. 1:**
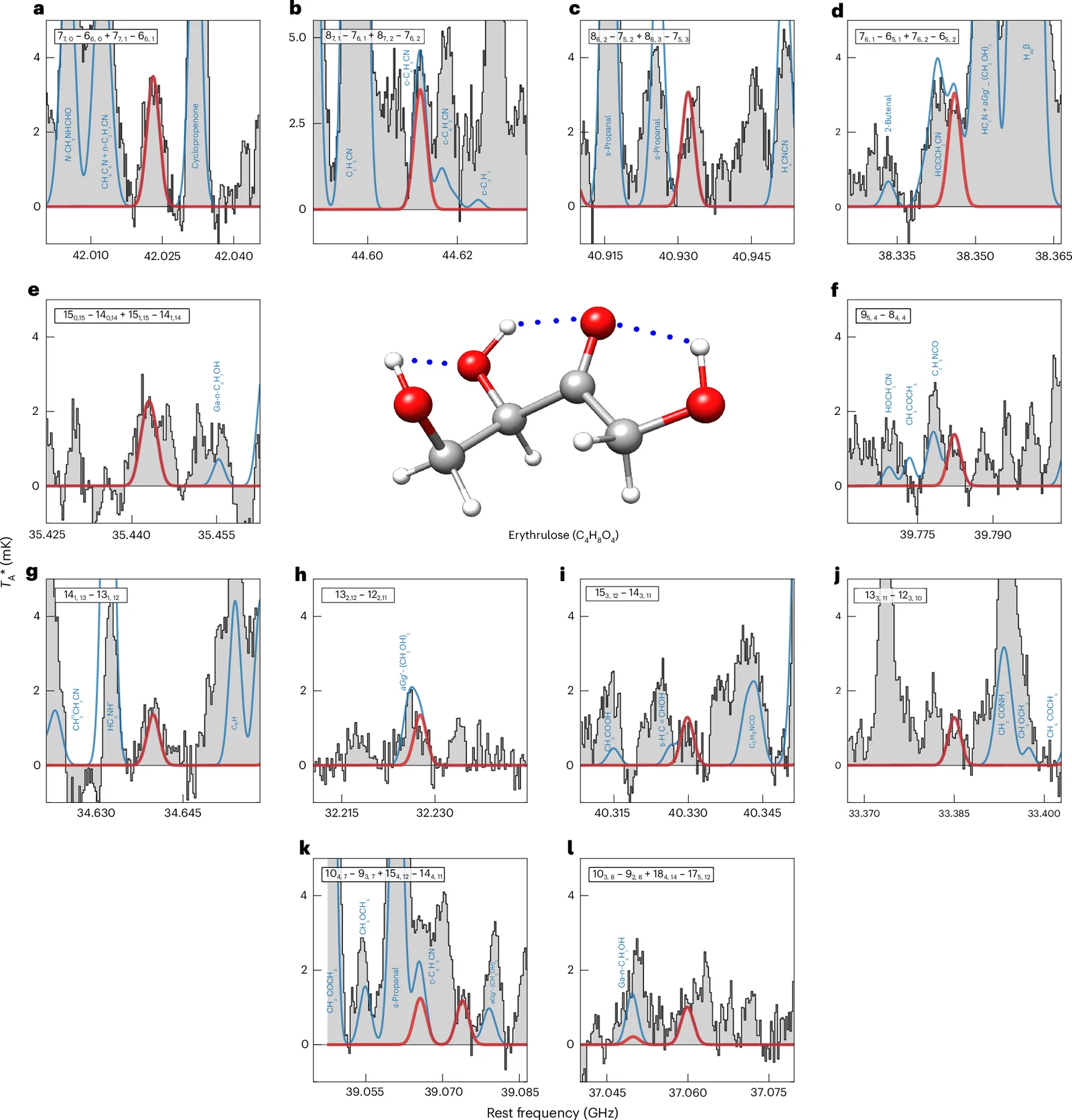
Brightest and most unblended transitions of erythrulose observed towards the G+0.693 molecular cloud. **a**–**l**, Filled histograms report the observed spectra, red lines show the line profiles of the erythrulose transitions fitted with MADCUBA-SLIM, and blue lines present the total fit to the spectra considering all the molecules identified towards the cloud. The intensity of the observed spectra is shown in units of antenna temperature, *T*_A_*. The quantum numbers of each transition of erythrulose are given in the upper part of each graph. Blue labels indicate the molecular species contributing to the observed spectra in the vicinity of the erythrulose lines. The transitions are sorted from the brightest to the weakest lines according to the LTE model.

Although line blending remains a challenge in chemically rich sources such as G+0.693, the analysis presented here identifies a set of transitions that are consistent with the predicted spectrum of erythrulose. Figure 1 shows 12 sets of lines of erythrulose (accounting for a total of 17 individual transitions) identified in the G+0.693 cloud using the MADCUBA-SLIM software (https://cab.inta-csic.es/madcuba/; see also Extended Data Table 1). These correspond to the brightest and most unblended transitions of erythrulose present in our dataset with integrated intensities ≥9*σ*—with $\sigma \,=\,\mathrm{r.m.s.}\times \,\sqrt{\delta v\times {\rm{FWHM}}}$, where r.m.s. is the root mean square noise level of the spectra, δ*v* is the velocity resolution and FWHM the full width at half maximum of the lines—which are used to fit the erythrulose emission under the assumption of local thermodynamic equilibrium (LTE) using MADCUBA-SLIM (Methods). The individual contribution of the erythrulose transitions is shown in red, while in blue we report the total spectrum predicted by MADCUBA-SLIM considering the contribution from all molecular species identified and modelled towards G+0.693 (more than 180 species, including isotopologues, are considered in the model). Note that transitions from higher vibrational states are not expected in this cloud because the molecular emission is subthermally excited with measured *T*_ex_ ≤ 15 K (refs. ^34,37^).

Six sets of transitions out of the 12 shown in Fig. 1 are classified as predominantly unblended (at 42,023, 40,932, 35,442, 39,782, 40,329 and 33,385 MHz), with contamination levels ≤25% (Extended Data Table 1; the criteria to establish the predominantly unblended features of erythrulose are described in the Methods and Supplementary Information). This ensures that the emission in these features is dominated by erythrulose and provides a robust basis for identification. The transitions at 34,639, 39,073 and 37,060 MHz are classified as blended with U-lines according to our selection criteria (Methods; Extended Data Table 1). However, a visual inspection shows that the erythrulose fit predicted by MADCUBA-SLIM reproduces well the observed spectra (Fig. 1). The features at 44,611, 38,346 and 32,227 MHz are blended with known molecular species, but the agreement with the global fit is excellent, further reinforcing the detection of erythrulose.

**Extended Data Fig. 1:**
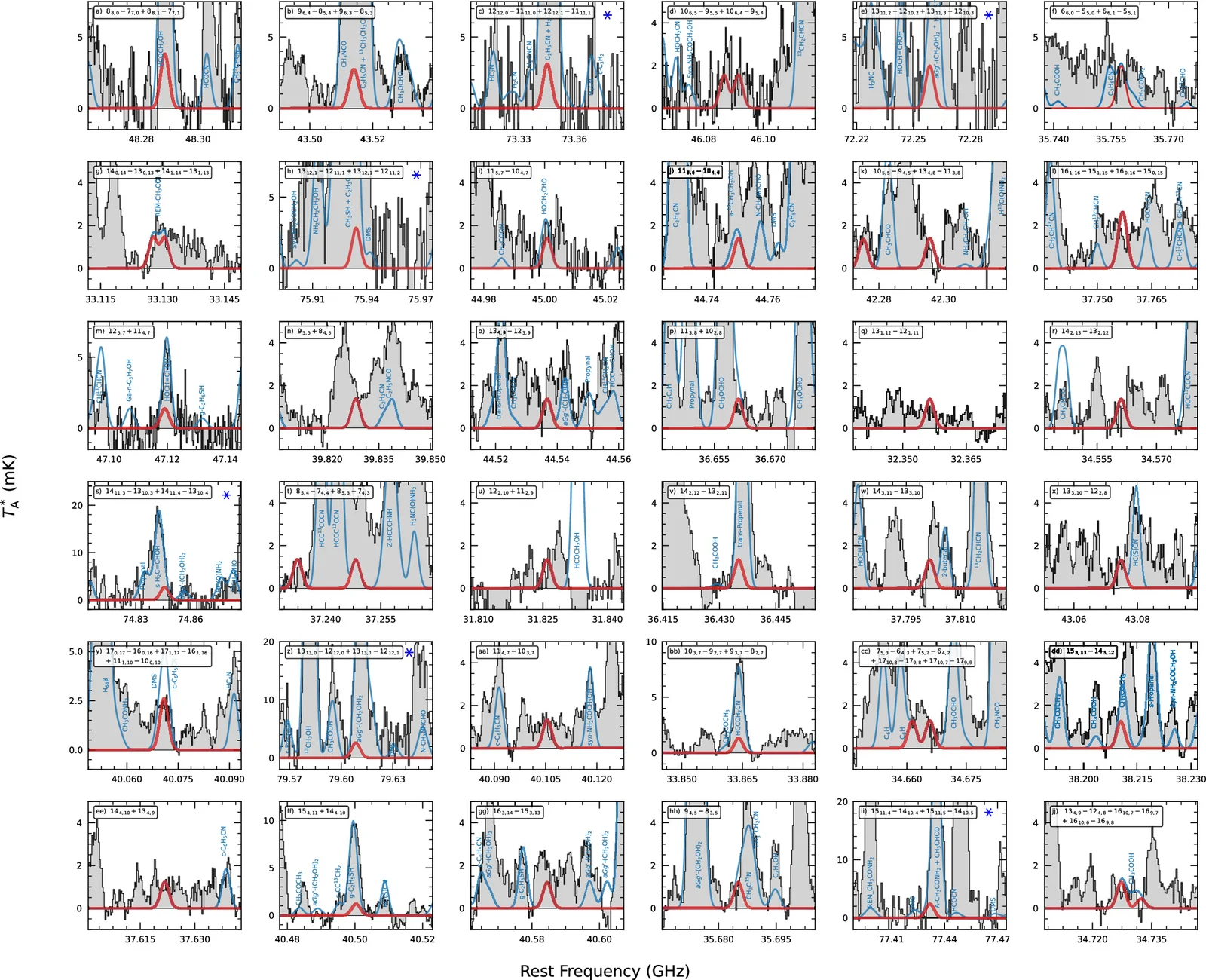
Remaining erythrulose transitions present in our dataset with peak intensities ≥1.2 mK. Filled histograms report the observed spectra, red lines show the line profiles of the erythrulose transitions fitted with MADCUBA-SLIM, and blue lines present the total fit to the spectra considering all the molecules identified toward G+0.693. The quantum numbers of each transition of erythrulose are given in the upper part of each panel. In blue, we label the molecular species contributing to the observed spectra in the vicinity of the erythrulose lines. Blue asterisks indicate those transitions covered within the 3mm atmospheric window.

Extended Data Fig. 1 shows the remaining transitions of erythrulose present in our dataset with peak intensities ≥1.2 mK, that is, 3× the average r.m.s. noise level in the spectra shown in Fig. 1. These transitions are weaker or more affected by blending and/or noise and are hence not used for the LTE fit of erythrulose. Despite not being used for the LTE fit, all these erythrulose lines predicted by MADCUBA-SLIM are consistent with the observed spectra. Given the physical properties of erythrulose in G+0.693 (see below), its brightest lines are expected to fall within the frequency range covered by Yebes 40 m (31–50 GHz), as observed for other complex organic molecules (COMs)^38^. However, some transitions also appear at 3 mm (Extended Data Fig. 1, blue asterisks), although they are largely blended. The agreement across the full set of detected transitions provides strong, independent validation of the identification.

The fit of the 12 sets of erythrulose transitions shown in Fig. 1 using the MADCUBA-SLIM tool yields an excitation temperature of *T*_ex_ = 11.3 ± 1.8 K and a column density of *N* = (8.7 ± 0.8) × 10^13^ cm^−2^ for a fixed central radial velocity *v*_LSR_ = 69 km s^−1^ and a linewidth FWHM of 22 km s^−1^ (Methods). The derived column density of (8.7 ± 0.8) × 10^13^ cm^−2^ translates into an erythrulose abundance of (6.4 ± 0.6) × 10^−10^ assuming an H_2_ column density of 1.35 × 10^23^ cm^−2^ for G+0.693 (ref. ^39^). Glycolaldehyde (with 2 C atoms) is observed with an abundance in G+0.693 similar to that of erythrulose (Table 1). By contrast, the only C3 sugars, glyceraldehyde and dihydroxyacetone, are not detected in this cloud, with upper limits to their abundance ≤4 × 10^−11^ and ≤7 × 10^−11^, respectively. Erythrulose appears to be ≥8–17 times more abundant than C3 sugars (Table 1). This is striking, as other chemical families (for example, alcohols, thiols, aldehydes and isocyanates) show an abundance decrease of roughly one order of magnitude with each added carbon atom^35,40–42^. One might consider the triol compound glycerol (HOCH_2_CH(OH)CH_2_OH) as a potential precursor of erythrulose, but this molecule was not detected in our survey either (Table 1).

**Table 1:** Derived physical parameters and modelled abundances of erythrulose and chemically related species towards the G+0.693 molecular cloud

Molecule	*N*	*T*_ex_	*v*_LSR_	FWHM	${\chi }_{\mathrm{obs}}^{{\rm{a}}}$	${\chi }_{\mathrm{mod}}^{{\rm{d}}}$
	(×10^13^ cm^−2^)	(K)	(km s^−1^)	(km s^−1^)	(×10^−10^)	(×10^−10^)
Glycolaldehyde (HOCH_2_CHO)^b^	9.3 ± 0.3	21.8 ± 0.8	68.9 ± 0.2	19.4 ± 0.5	6.9 ± 0.2	~20
aGg′-ethylene glycol [aGg′-(CH_2_OH)_2_]	23.5 ± 5.2	10.3 ± 2.6	67.9 ± 2.8	21^c^	17.4 ± 3.9	~100
Glyceraldehyde (HOCH_2_CH(OH)CHO)	≤0.5	10^c^	69^c^	21^c^	≤0.4	~20
Dihydroxyacetone (HOCH_2_COCH_2_OH)	≤1.0	10^c^	69^c^	21^c^	≤0.7	~50
Glycerol (HOCH_2_CH(OH)CH_2_OH)	≤2.5	10^c^	69^c^	21^c^	≤1.9	∼1
Erythrulose (HOCH_2_CH(OH)COCH_2_OH)	8.7 ± 0.8	11.3 ± 1.8	69^c^	22^c^	6.4 ± 0.6	~20

^a^Molecular abundances calculated using *N*(H_2_) = 1.35 × 10^23 ^cm^−2^ (ref. ^39^).

^b^Derived physical parameters taken from ref. ^9^.

^c^This parameter was fixed in MADCUBA SLIM-AUTOFIT to reach convergence.

^d^Modelled abundances at 1.6 Myr from the simulation with *ζ* = 1.3 × 10^−14^ s^−1^.

We can estimate the confidence level of the erythrulose detection in G+0.693 following refs. ^43,44^. Assuming the confusion limit and Gaussian line profiles, the chance of finding a feature at the *v*_LSR_ of G+0.693 with a tolerance of ±4 km s^−1^ (that is, the largest velocity shift observed for the molecular line emission in G+0.693)^35,45^ is *p* = 36% for a velocity coverage equivalent to the FWHM of the erythrulose lines. The detection of each successive line has a probability *p*^*n*^, where *n* is the number of unblended transitions. The probability of chance alignment for the six most unblended transitions is then *p*^6^ = 0.2%, providing strong statistical support for the identification of erythrulose. It is important to stress that, although G+0.693 presents line-rich and complex spectra, the molecular emission in this cloud is subthermally excited with low *T*_ex_, which yields much lower levels of line blending and line confusion as compared to hotter sources such as massive hot cores and low-mass hot corinos. Even if three or four unblended lines were considered in our analysis, we would still obtain confidence levels of 95.2% and 98.3%, respectively. Note that this estimate is conservative because line confusion has not been reached in our G+0.693 spectral surveys.

### Formation mechanism on interstellar ice surfaces

The C3 sugars glyceraldehyde and dihydroxyacetone are at least 8–17 times less abundant than erythrulose, suggesting that they are unlikely to be its dominant precursors under the physical conditions of G+0.693. Therefore, we turn our attention to smaller and more abundant building blocks of this C4 sugar such as glycolaldehyde and ethylene glycol. Recent laboratory experiments of irradiated CH_3_OH ices reveal that sugars with up to six carbon atoms can be produced from smaller sugar and sugar-derivative fragments (that is, C6 sugars would form from C3 sugar precursors)^46^. By inspecting the chemical structure of erythrulose, one can envision its formation via the combination of two C2 fragments: CH_2_OHC^•^O and C^•^HOHCH_2_OH. The first fragment is the glycolaldehyde radical (g*), and the second is the ethylene glycol radical (e*), which contains a pro-chiral centre at carbon C^•^, responsible for the chirality of erythrulose. Both glycolaldehyde and ethylene glycol are present in G+0.693 at high abundances; their measured abundances are (6.9 ± 0.2) × 10^−10^ and (1.7 ± 0.4) × 10^−9^, 1.1 and 2.7 times the erythrulose abundance, respectively (Table 1). Moreover, the radical g* has been identified as the main intermediate species formed in laboratory experiments simulating the reaction of atomic H with glycolaldehyde in solid para-H_2_ (dense cloud conditions)^47^. Besides, sugar acids have also been synthesized experimentally by the energetic processing of interstellar ice analogues containing ethylene glycol and CO_2_, through the formation of the radical e* (ref. ^48^). Therefore, the combination of g* and e* could lead to the synthesis of erythrulose in the ISM.

**Fig. 2:**
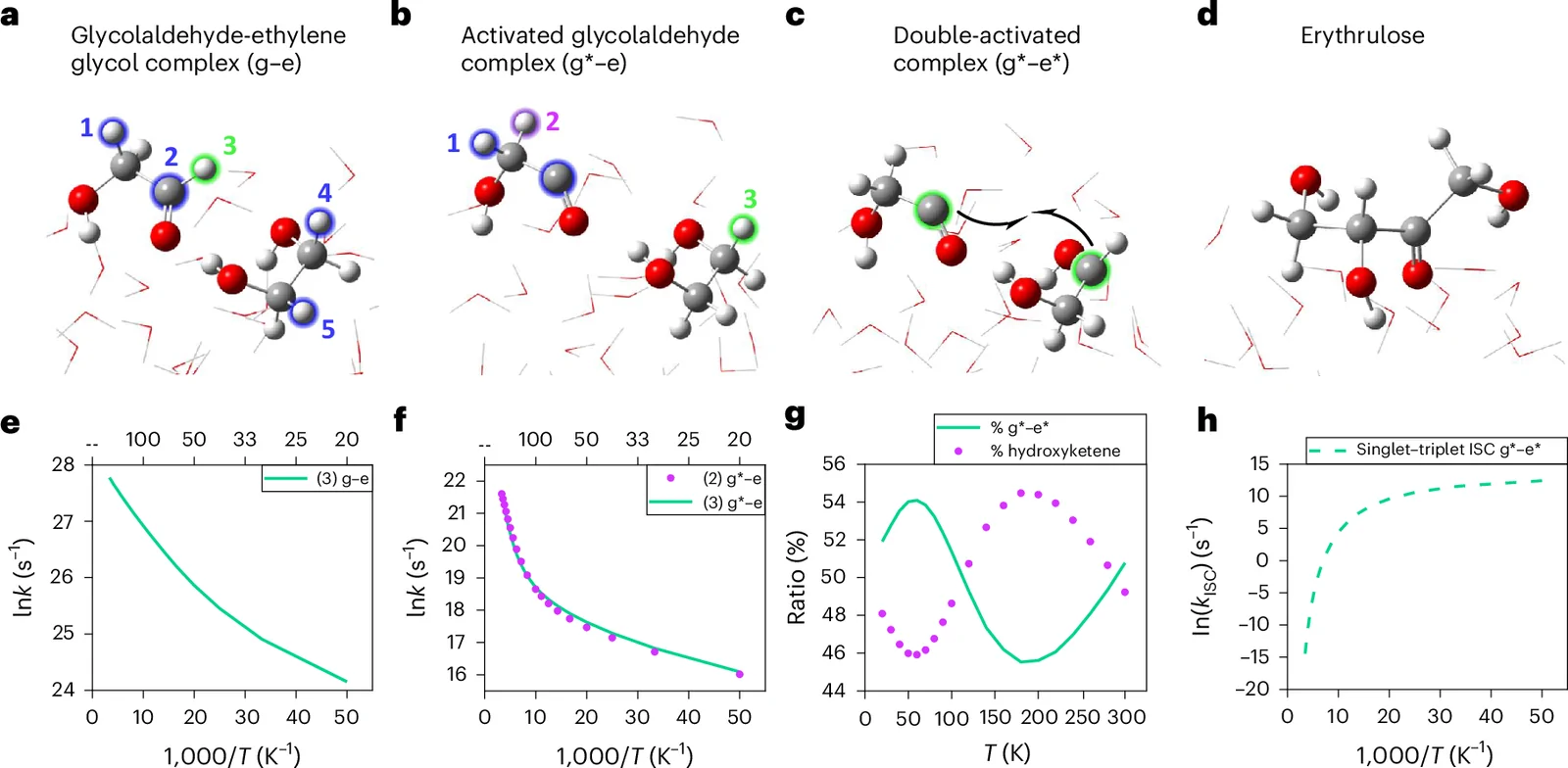
Reaction mechanism of erythrulose from glycolaldehyde (g) and ethylene glycol (e) on ASW. **a**–**d**, Optimized geometries are shown for the complexes g–e (**a**), the first activated g*–e complex (**b**), the doubly activated g*–e* complex (**c**) and erythrulose (**d**). The available hydrogen and carbon atoms for abstraction, addition and recombination reactions are highlighted in blue, green and purple. The green and purple highlighted hydrogen atoms in **a** and **b** represent, respectively, the most viable reactions. Green carbon atoms in **c** represent the recombination of the two radicals. **e**,**f**, Arrhenius plots of the thermal rate constants for reaction 3-g–e (**e**) and reactions 2-g*–e and 3-g*–e (**f**). **g**, The branching ratios for reactions 2-g*–e and 3-g*–e leading to the complexes hydroxyketene–ethylene glycol (not shown) and g*–e*. **h**, The rate constant for the ISC of the g*–e* complex. Some water molecules have been removed from the images of the optimized geometries for the sake of clarity.

Figure 2 illustrates the formation mechanism of this C4 ketose sugar on the surface of amorphous solid water ice (ASW) from the radicals g* and e*. This formation mechanism has two isoenergetic mirror symmetry mechanisms that form the two enantiomers with 50% probability each. Figure 2a shows the most favourable orientation of the glycolaldehyde–ethylene glycol complex (g–e) on the ASW and all possible reactive collisions of atomic H with the complex. Among these, the hydrogen abstraction reaction 3-g–e, is the fastest reaction (Fig. 2a, green), with an energy barrier of 4.1 kJ mol^−1^ and a reaction energy of 27.4 kJ mol^−1^ (zero-point energy corrected; see the Supplementary Information for further details). The quantum tunnelling-corrected rate constant of reaction 3-g–e ranges from 3.1 × 10^10^ s^−1^ to 1.2 × 10^12^ s^−1^ in the temperature range of 20–300 K (Fig. 2e), and hence it is a very fast reaction.

After the H abstraction reaction 3-g–e, the activated glycolaldehyde complex (g*–e; Fig. 2b) is formed, which could proceed with either subsequent H abstraction reactions or two isomerization reactions by hydrogen migration to the activated carbon of the aldehyde group. The two isomerization reactions present energy barriers of 77.0 and 97.3 kJ mol^−1^, which makes them non-viable under ISM conditions. For the H abstraction reactions, reaction 1-g*–e shows a repulsive potential (Supplementary Information). However, reactions 2-g*–e and 3-g*–e are energetically favoured and form hydroxyketene for reaction 2-g*–e, and the doubly activated complex g*–e* for reaction 3-g*–e (Fig. 2c). The branching ratios for these two reactions are approximately 50% in the whole range of temperatures, being slightly higher for the formation of the g*–e* complex at low temperatures (Fig. 2g). The tunnelling-corrected rate constant of reaction 3-g*–e ranges from 9.7 × 10^6^ s^−1^ to 2.5 × 10^9^ s^−1^ between 20 K and 300 K.

Once formed, the g*–e* complex is in the triplet state and the radicals cannot recombine because the approach of atoms having parallel electrons induces a repulsive potential. However, the g*–e* complex can experience a spin change under ISM conditions throughout an intersystem crossing (ISC) process, because both states are practically degenerate with an energy difference (zero-point energy corrected) of only 0.04 kJ mol^−1^ in favour of the singlet state (Supplementary Information). The latter process shows rate constants of 2.4 × 10^5^ s^−1^–1.6 × 10^−7^ s^−1^ between 20 K and 300 K, making it the slowest step in the entire reaction mechanism (Fig. 2h and Supplementary Table 2). Finally, the association of the two carbons with antiparallel spin yields erythrulose (Fig. 2d).

### Astrochemical modelling of erythrulose formation

**Fig. 3:**
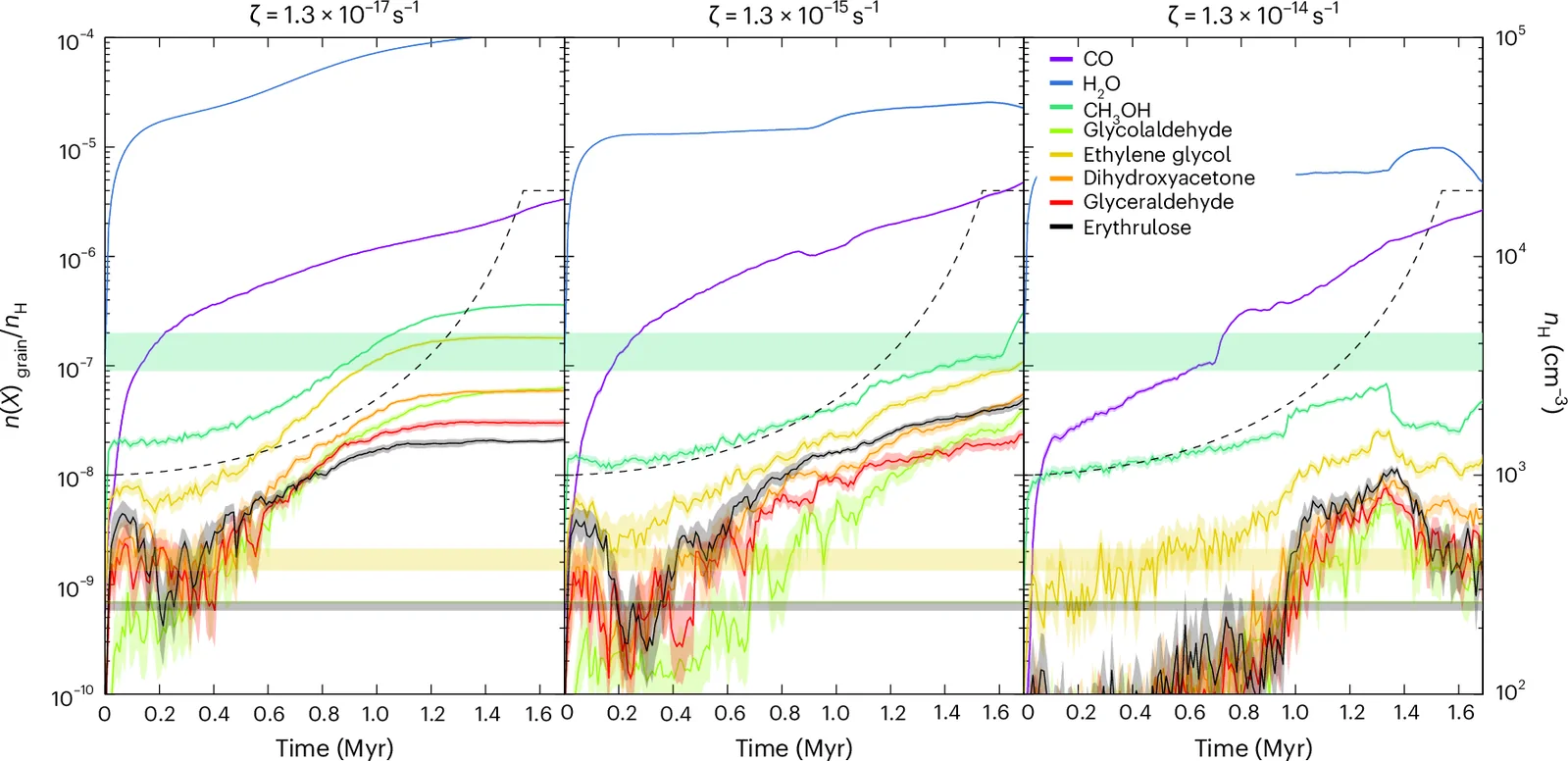
KMC simulations of the ice build-up in a cloud with similar physical conditions to those of G+0.693. Colour lines indicate the solid ice abundances of CO, H_2_O, CH_3_OH, glycolaldehyde, ethylene glycol and all C3 and C4 sugars considered in the model (Methods). The ratio *n*(*X*)_grain_/*n*_H_ refers to the molecular fractional abundance measured with respect to hydrogen nuclei, *n*_H_. *n*(*X*)_grain_ is the volume density of molecule *X* in the ice in units of cm^−^^3^. Data are presented as mean values ± 1 standard deviation spread of the nine simulations carried out with different random seeds. Labels are shown on the right, and the cosmic-ray ionization rate, *ζ*, is presented at the top of each panel. Dashed lines indicate the increase of the gas density during the cloud’s collapse. Horizontal colour-shaded ranges show the molecular abundance values measured towards G+0.693 together with their 1*σ* uncertainty (Table 1).

We have demonstrated that erythrulose can form on the surface of interstellar dust grains from glycolaldehyde and ethylene glycol at a rate constant of (1–2) × 10^5^ s^−1^ at the typical dust temperatures measured in Galactic Centre molecular clouds (*T*_dust_ ≈ 20–30 K)^49,50^. Using this information, we now evaluate whether we can predict the observed amount of erythrulose in the G+0.693 cloud by implementing the mechanism described above in a kinetic Monte-Carlo (KMC) simulation code^51–54^. We expanded our chemical network to include the formation of other C3 and C4 sugars (glyceraldehyde, dihydroxyacetone, threose and erythrose) and related compounds (for example, glycerol). The network allows radicals from C1 and C2 species (for example, H^•^CO, H_2_C^•^OH, HC^•^OHCHO, CHOHC^•^O and CH_2_OHC^•^HOH) to recombine^54^, forming five different C3 compounds (including the sugars dihydroxyacetone and glyceraldehyde, plus glycerol and two C3 aldehydes) and six different C4 compounds (including threose and erythrulose). Additional hydrogen abstraction, hydrogenation and ultraviolet (UV) photodissociation reactions (including those inducing the breaking of the CC bond of glycolaldehyde and ethylene glycol) were added to allow the interconversion between species (Methods). We ran simulations of the ice build-up for different cosmic-ray ionization rates: *ζ* = 1.3 × 10^−17^ s^−1^ (the standard Galactic value) and 1.3 × 10^−15^ s^−1^ and 1.3 × 10^−14^ s^−1^ (100 and 1,000 times higher, as applicable to Galactic Centre molecular clouds^55,56^). The model assumes an initial hydrogen-nuclei density of 10^3^ cm^−3^, rising to 2 × 10^4^ cm^−3^ over 1.5 Myr during the collapse of the cloud, after which the density is kept constant (Fig. 3). We fix the dust temperature at *T*_dust_ = 20 K, consistent with observations of G+0.693 (ref. ^50^). Each simulation was run with nine different random seeds.

Figure 3 shows that both C3 and C4 sugars can form efficiently in the ice in our models. Erythrulose is the most efficiently produced C4 sugar, achieving higher abundance than the C4 aldoses (threose and erythrose) under most conditions. Consistent with our observations, erythrulose tends to reach slightly higher abundances than the C3 sugars (glyceraldehyde and dihydroxyacetone), especially at higher *ζ* and shorter timescales. This behaviour results from the higher UV photodestruction rate assumed for C3 sugars compared with C4 sugars (Methods). Chemically, smaller sugar molecules are expected to be more easily destroyed than larger ones, as larger molecules have more degrees of freedom to distribute energy upon UV absorption. In our simulations, ethylene glycol is always more abundant than any C3 and C4 sugar, in agreement with our observations. Glycolaldehyde, however, reaches abundances comparable to, or slightly higher than, those of the C3 and C4 sugars only towards the end of the simulations.

Molecular clouds in the Galactic Centre are pervaded by low-velocity shocks from cloud shearing and collisions^57^. G+0.693 is believed to be undergoing such a collision, driving a large-scale shock of *v*_s_ ≈ 20 km s^−1^ (ref. ^37^). Ice material is thus expected to be partially injected into the gas phase in G+0.693 by grain sputtering^58^. Figure 3 shows that the KMC models whose ice abundances come closest to the observed gas-phase values in G+0.693 are those with *ζ* ≥ 1.3 × 10^−14^ s^−1^. Such high values of *ζ* were also invoked in previous models of this source to explain the abundances of certain molecular ions (for example, PO^+^ and HOCS^+^)^59,60^. In our simulations, the predicted abundances in the ice of CH_3_OH, glycolaldehyde, ethylene glycol and erythrulose all lie within a factor of 5 of the observed values (Table 1) and well within the tolerance of one order of magnitude typically considered in chemical modelling. The C3 sugars (glyceraldehyde and dihydroxyacetone) are overproduced in the model by factors of ~25–70. However, the abundance ratios of glycolaldehyde, ethylene glycol, and the C3 sugars with respect to erythrulose differ by slightly more than one order of magnitude (Table 2), while remaining broadly consistent given the large uncertainties in our simulations.

**Table 2:** Observed and predicted abundance ratios with respect to erythrulose

Abundance ratio	Observed	Modelled	Observed
			Modelled
Glycolaldehyde/erythrulose	1.1	~1	~1
Ethylene glycol/erythrulose	2.7	~5	~0.5
Erythrulose/glyceraldehyde	≥17.4	~1	≥17
Erythrulose/dihydroxyacetone	≥8.7	~0.4	≥22

The predicted abundances are taken from the model with *ζ* ≥ 1.3 × 10^−14^ s^−1^ at ~1.6 Myr.

The discrepancy between the modelled abundances in the ice and the observed abundances in the gas could be due to a combination of effects. First, only a fraction of the ices may be sputtered in the shock (≤30%, based on the KMC results). C3 and C4 sugars are more refractory than water and, hence, may be harder to eject from grains. Alternatively, C3 and C4 sugars may rapidly re-adsorb onto dust grains given the low dust temperature (*T*_dust_ = 20 K) in G+0.693. This could result in less of those sugars remaining in the gas to be observed. The detection of CO and CO_2_ ice in Galactic Centre molecular clouds^61^ indicates that a fraction of the ices remain on dust grains despite the dust energetic processing. In addition, note that the KMC simulations do not include an extended gas-phase chemical network for these COMs and, hence, C3 and C4 sugars may undergo efficient gas-phase destruction through ion-neutral reactions^60^ after the ice release in the shock. Glyceraldehyde has recently been found to be more unstable than glycolaldehyde in the gas phase, which may explain its lack of detection^62^. Finally, uncertainties in reaction rates (for example, photodissociation efficiencies)^63^, reaction branching ratios or even unmodelled processes might also play a role in overestimating sugar abundances in the ice in our model.

## Discussion

Erythrulose, with 14 atoms in its structure, represents the largest non-cyclic molecular species identified so far in the ISM (cf. ref. ^64^), and the first detected molecule containing four oxygen atoms (cf. the catalogue in ref. ^1^). It is also the first sugar and the second chiral molecule reported in the ISM after that reported in ref. ^65^. Its detection not only provides direct evidence that complex, chiral species can form under interstellar conditions, but it also takes us to a higher level in the ladder of interstellar chemical complexity, suggesting that other prebiotic (and potentially chiral) molecules could also form and survive under the extreme conditions of the ISM.

Prebiotic chemistry experiments have shown that ribonucleotides—the building blocks of RNA—can be synthesized from mixtures containing sugars such as erythrulose^66^. Ketoses such as erythrulose can readily isomerize into their aldose counterparts (that is, threose and erythrose) in aqueous environments^67^, a transformation that is central to the formation of ribose and then nucleic acids^68^. However, prebiotic experiments have typically introduced such sugars as external inputs. Our detection of erythrulose in the G+0.693 molecular cloud demonstrates that this monosaccharide can be synthesized abiotically under interstellar conditions. The similarity of the organic inventories of comets and presolar environments supports a connection between the chemistry observed in the ISM and that inherited by minor bodies of the Solar System^69^. Therefore, the detection of interstellar erythrulose suggests that complex sugars may have formed in the protosolar molecular cloud and were subsequently transferred to minor bodies.

Sugars such as ribose and glucose have indeed been found in meteorites^20^ and in asteroid Bennu^21^. In addition, erythrulose has also been proposed to exist on the surface of outer Solar System bodies, including the Kuiper Belt object Arrokoth^46^. Laboratory irradiation experiments support the interstellar formation of erythrulose, as well as other sugar derivatives such as sugar acids and sugar alcohols^46,48^, in agreement with our astrochemical model. Our astrochemical simulations indeed show that erythrulose can form efficiently in ices under a range of cosmic-ray ionization rates, including those expected in dust traps of protoplanetary disks^70^. In these regions, energetic processing transforms simple ices into macromolecular organics, with up to 4% of the ice reservoir affected^70^. Hence, once formed, erythrulose and other sugars may be incorporated into planetesimals (as measured in Arrokoth^46^ and meteorites^20^), later contributing to the inventory of prebiotic organic material on planetary surfaces. The discovery of chiral erythrulose also supports the idea that small enantiomeric excesses measured in meteorites (of a few per cent)^71,72^ may originate in extraterrestrial environments, which could have contributed to the emergence of biological homochirality on early Earth through subsequent chemical amplification processes^73^.

Even under conservative assumptions—considering only the observed gas-phase abundance of erythrulose in G+0.693—and given the interstellar water abundance of 10^−4^ (see ref. ^74^), the erythrulose/water abundance ratio measured in G+0.693 is of ~6 × 10^−6^. Other prebiotic organics such as ethanolamine, also detected in G+0.693, have been found in meteorites with abundance ratios with respect to water similar to those measured in the ISM^8^. Therefore, considering that the average water content in meteorites is 7,500 ppm (ref. ^75^) and an estimated amount of organic matter delivered to early Earth of 10^16^–10^18^ kg (ref. ^76^), we estimate that ~(0.5–50) × 10^9^ kg of erythrulose could have been delivered to early Earth during the Late Heavy Bombardment between 4.1 and 3.9 billion years ago^77^. Although the extent and intensity of the Late Heavy Bombardment has recently been questioned^78^, Monte-Carlo simulations of Earth’s impact history show that biopoiesis—the process describing the development of living matter from non-living organic matter—could occur within a much broader timeline between 4.45 and 3.9 billion years ago with at least a 16% chance^79^. Because glycolaldehyde, small sugars and sugar derivatives have been shown to survive meteoritic impacts^80,81^, the presence of erythrulose in interstellar space strengthens the case for an exogenous origin of sugars relevant to the synthesis of the first nucleic acids. The presence of erythrulose on the surface of a primitive Earth and its rapid isomerization into threose^67^ support its involvement in the formation of threose nucleic acid (TNA), a structurally simpler RNA analogue and the simplest of all potential sugar-containing nucleic acids. Threose nucleic acid has been synthesized in the laboratory and, in the context of the origin of life, has been proposed as one of the polymers that could have been involved in a pre-RNA world^82^. Therefore, the discovery of interstellar erythrulose suggests that the ISM could be a viable source of sugar feedstock for the prebiotic synthesis of the first nucleic acids, not only on the primitive Earth but also elsewhere in the Universe.

## Methods

### Observational campaign and spectral coverage

We conducted a broadband, ultrasensitive spectral survey of the Galactic Centre molecular cloud G+0.693 using the Yebes 40 m (Guadalajara, Spain) and IRAM 30 m (Granada, Spain) radiotelescopes. Observations were performed in position-switching mode towards *α*(J2000) = 17 h 47 min 2 s, *δ*(J2000) = −28° 21′ 27″, with *α* the right ascension and *δ* the declination of the source, and using an off position shifted by Δ*α* = −885″ and Δ*δ* = 290″. The Yebes 40 m data (project 21A014) were collected between March 2021 to March 2022 using the ultrabroadband Nanocosmos Q band (7 mm) HEMT receiver, which provides full frequency coverage from 31.07 to 50.42 GHz in two linear polarizations^83^. Spectra were recorded using the 16 fast Fourier transform spectrometers (38 kHz channel width), with frequency set-ups at 41.4 and 42.3 GHz to flag spurious features. Data reduction and averaging were performed using the Python-based pipeline (https://github.com/andresmegias/gildas-class-pipeline/) developed by ref. ^84^ and the MADCUBA software (https://cab.inta-csic.es/madcuba/index.html; see ref. ^85^). The final spectra were smoothed to a frequency resolution of 256 kHz (1.5–2.5 km s^−1^). The half power beam width of the telescope ranged between 35″ and 55″. All data were calibrated in ${T}_{{\rm{A}}}^{* }$ units because the molecular emission towards G+0.693 is extended over the beam^33,34^. The final r.m.s. noise achieved was 0.25–0.9 mK per channel.

The IRAM 30 m observations (projects 123-22 and 076-23) were performed during different sessions in 2023 (February) and 2024 (March and April) using the Eight Mixer Receivers (EMIR). Spectral coverage spanned 83.2–115.41, 132.28–140.39 and 142–173.81 GHz. Each frequency set-up was shifted in frequency to identify possible contamination of spurious lines coming from the image band. The fast Fourier transform spectrometer (FTS200) provided a spectral resolution of 195 kHz, although the spectra were subsequently smoothed to 615 kHz (1.0–2.2 km s^−1^). The spectra were calibrated in ${T}_{{\rm{A}}}^{* }$ units. The half power beam width ranged from 14″ to 29″. The final r.m.s. noise was 0.5–2.5 mK at 3 mm and 1.0–1.6 mK at 2 mm per channel. Frequency gaps were filled using data from previous IRAM surveys^36,40^.

### Identification of predominantly unblended transitions

The rotational spectrum of the L-1 conformer of erythrulose—the lowest-energy open-chain conformer—was measured in the laboratory by ref. ^28^. Its spectroscopic entry was calculated using SPCAT up to 720 GHz. Predicted frequencies below 18 GHz have uncertainties as low as 1 kHz, which translates into errors of up to 120 kHz in the Q band, equivalent to ~0.7–1.2 km s^−1^. These uncertainties are much smaller than the typical linewidths (of ~20 km s^−1^) observed in G+0.693 and than the spectral resolution of our data (minimum of 256 kHz). A similar approach has been used for the discovery of several other molecular species in this cloud, even when using spectra at higher frequencies (for example, at 3 mm; see the case of HOCS^+^)^60,86^.

The SPCAT spectroscopy entry was incorporated into the MADCUBA package (version 31/05/2024)^85^, and transitions of erythrulose were identified using the Spectral Line Identification and Modeling (SLIM) tool under the assumption of LTE. SLIM generates the LTE synthetic spectra for comparison with observed data. This allows us to identify the brightest and most unblended transitions of erythrulose shown in Fig. 1. The line classification has been carried out following the criteria described in ref. ^87^, which are based on the accepted standard of ref. ^43^ and ref. ^44^:

1. Accurate rest frequencies: The frequency uncertainties of the identified erythrulose transitions are ≤120 kHz (see values in parentheses within the first column of Extended Data Table 1), which lies below the spectral resolution of our Yebes 40 m and IRAM 30 m data with a minimum value of 256 kHz.
2. Frequency agreement: “An accurate astronomical rest frequency of the assigned transition must be in reasonable agreement with the frequency corresponding to the LSR velocity of the source”^43^. The erythrulose emission is well reproduced by fixing its *V*_LSR_ to 69 km s^−1^ (Table 1), in excellent agreement with the *V*_LSR_ obtained for other COMs towards G+0.693 (ref. ^34^).
3. Linewidth agreement: The derived linewidth of the erythrulose lines is 22 km s^−1^ (Table 1). This value is consistent with those observed for other molecules towards the G+0.693 cloud^88^.
4. Beam dilution: Our data do not suffer from beam dilution because the emission of COMs such as glycolaldehyde and ethylene glycol towards G+0.693 is extended across the Sgr B2 molecular cloud^89^ and, therefore, across the single-dish beams of the IRAM 30 m and Yebes 40 m telescopes.
5. Relative intensities: “Once several molecular transition assignments have been made, their relative intensities must be tested for consistency”^43^. All observed spectral features are consistent with the LTE predictions obtained with MADCUBA-SLIM (Fig. 1 and Extended Data Fig. 1).
6. Confirmation of transitions: “There must be spectral features at all favorable, physically connected transitions over a sufficiently large wavelength range”. Our dataset covers over 91 GHz in the 7 mm, 3 mm and 2 mm wavelength ranges, and all predicted features of erythrulose are consistent with the observed spectra. Note that the global LTE fit overpredicts the spectrum for the 17_0,17_ → 16_0,16_ and 17_1,17_ → 16_1,16_ transitions at ~40.070 GHz (Extended Data Fig. 1y), but this can be considered an outlier in our model. Other blended transitions such as the 12_5,7_ → 11_4,7_ and 15_4,11_ → 14_4,10_ transitions (Extended Data Fig. 1m,ff), and the 14_11,3_ → 13_10,3_+14_11,4_ → 13_10,4_ and 10_3,7_ → 9_2,7_+9_3,7_ → 8_2,7_ line sets (Extended Data Fig. 1s,bb), match the observed spectra very well.
7. Level of blending: To determine the level of blending with other molecules or U-lines, we obtained the residual area after subtracting the LTE fit of the erythrulose lines from the total measured area in the observed spectra, over the velocity range defined by *V*_LSR_ = 69 ± FWHM (that is, over 2 × FWHM). This velocity range covers 98.2% of the total area underneath a Gaussian line profile. As shown in Supplementary Fig. 2, if one considers the most unfavourable case in which the observed spectral feature is flat due to heavy line blending, the derived residual area would represent ~50% the total area measured over a velocity range of 2 × FWHM. Therefore, we establish a criterion whereby an observed feature is considered mostly unblended if the residual contribution is less than half this limit, that is, ≤25% (Supplementary Information). A similar approach has been adopted in recent detections of new molecules in G+0.693, including large complex organics^38,87^.

Following these criteria, we identify a total of six mostly unblended features, which account for nine individual transitions of erythrulose. Although blended with residual emission ≥25%, the visual inspection of Fig. 1b,d,h shows that the global LTE fit reproduces well the observed spectra for the sets of transitions 8_7,1_ → 7_6,1_+8_7,2_ → 7_6,2_, and 7_6,1_ → 6_5,1_+7_6,2_ → 6_5,2_, and for the 13_2,12_ → 12_2,11_ line. All these transitions, together with the consistency of the remaining lines shown in Fig. 1 and in Extended Data Fig. 1, yield a robust assignment of erythrulose. Full line parameters of the transitions shown in Fig. 1 are listed in Extended Data Table 1.

### LTE analysis with MADCUBA

We performed a nonlinear least-squares LTE fit of the erythrulose emission by applying the AUTOFIT tool based on the Levenberg–Marquardt algorithm within SLIM^85^ to derive the best-fit physical parameters: excitation temperature (*T*_ex_), radial velocity (*v*_LSR_), linewidth (FWHM) and column density (*N*). To achieve convergence, the FWHM of the erythrulose emission had to be fixed to 22 km s^−1^, consistent with the typical line profiles in G+0.693 (refs. ^33,34^). Derived values are reported in Table 1 and agree with those obtained for other COMs in this source^33,34^.

### Computational methods

We simulated the ASW ice by carrying out molecular dynamic simulations of 100 water molecules at 300 K to amorphize the system, which was then cooled at 10 K to preserve the amorphous structure. Because our mechanistic approach involves the non-diffusive association of activated glycolaldehyde (g) and ethylene glycol (e) on ASW ice (Fig. 2), we placed several glycolaldehyde molecules around ethylene glycol and optimized each pair, obtaining only two stable structures: one with a carbonyl–hydroxyl interaction (CO) and another with a hydroxyl–hydroxyl interaction (OH). The two optimized CO and OH structures were used as fragments in a rigid docking study to generate glycolaldehyde–ethylene glycol complexes placed across the ASW surface (Fig. 2). Binding energies for both types were similar overall, but the CO complex showed a higher maximum interaction energy, so its strongest-bound structure was selected for the mechanistic study (Supplementary Fig. 3). Electronic structure calculations were performed with Gaussian 16^90^ using a three-layer ONIOM method (PW6B95(D3)/def2-TZVP:PM7R6:UFF)^91–94^ to model the reaction mechanism on ASW ice. Reactivity was initiated via hydrogen abstraction reactions forming radicals on the surface. The ISC needed to form erythrulose was studied using CASSCF(6e,5o)/def2-TZVP in ORCA^95,96^. The unimolecular rate constants for the hydrogen abstraction reactions were studied using transision state theory using the Pilgrim software^97^. Tunnelling corrections were applied using the Eckart approximation^98^. The ISC rate of the g*–e* intermediate was estimated using Marcus’ semi-classical theory^99,100^. The details of these calculations can be found in the Supplementary Information.

### Grain-surface chemistry simulations using the KMC approach

We extended a previously established grain-surface network^54,101^ to include six C3-forming radical-radical reactions (leading to, for example, tartonaldehyde, hydroxypyruvaldehyde, glyceraldehyde, dihydroxyacetone and glycerol) and seven C4-forming reactions (leading to 2,3-dihydroxybutanedial, 2,4-dihydroxy-3-oxobutanal, tetrose, dimethylolglyxoxal, erythrulose and tetritol). The rate constant is estimated to be 2 × 10^6^ s^−1^. Additional hydrogenation reactions and abstraction reactions (*k* = 10^5^ s^−1^) were included to allow interconversion via radical intermediates^102^. The C3 radicals can react with HC^•^O and C^•^H_2_OH to C4 sugar and sugar-derivative species. The glycolaldehyde–ethylene glycol complex was also included explicitly in the model to account for the surface reactions outlined in Fig. 2 (rate constants from Supplementary Table 2). All photodissociation processes were modelled using canonical rate constants of the following form.

For C2 species:

1 $$k=1{0}^{-9}\exp (-2.5{A}_{V})+500\zeta \,{{\rm{s}}}^{-1};$$

for C3 species:

2 $$k=9.0\times 1{0}^{-10}\exp (-2.5{A}_{V})+450\zeta \,{{\rm{s}}}^{-1};$$

for C4 species:

3 $$k=8.1\times 1{0}^{-10}\exp (-2.5{A}_{V})+405\zeta \,{{\rm{s}}}^{-1}.$$

The slightly reduced photodissociation rates for C4 species reflect their greater resilience due to higher internal degrees of freedom. The grain-surface chemistry is triggered by deposition of H, H_2_, C, N, O, CO, N_2_ and O_2_. Their fluxes are calculated self-consistently, assuming that these represent the main elemental reservoirs in the gas phase, with the remainder residing on grain surfaces. UCLCHEM (https://uclchem.github.io/v3.5.5/; see ref. ^103^) is used to determine the time-dependent gas-phase CO/C, O_2_/O and N_2_/N ratios and H and H_2_ fluxes.

## Supplementary information

Supplementary Information Supplementary Analysis and Methods, Figs. 1–7 and Tables 1 and 2.

Peer Review File

## Figures and Tables

**Extended Data Table 1:**
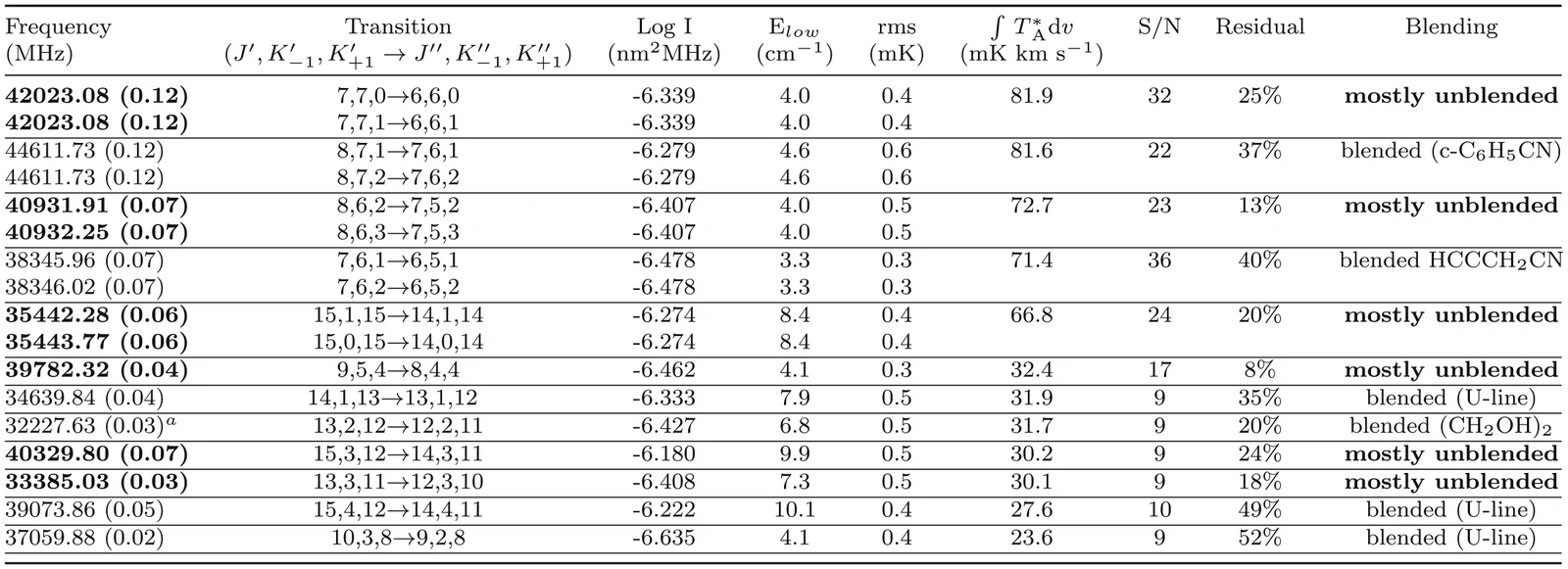
Spectroscopic information of the erythrulose transitions shown in Fig. 1

The S/N ratio is computed from the integrated signal $\int(T^*_{\rm{A}}\,{\mathrm{d}}v)$ and its associated noise level, $\sigma ={\rm{rms}}\,{\rm{x}}\sqrt{(\delta v\times {\rm{FWHM}})}$, where *δv* is the velocity resolution of the spectra and the FWHM is fitted from the data. We also provide the contribution of the contamination to the overall area after subtracting the LTE fit of erythrulose from the observed spectrum over a velocity range of ± FWHM (Residual area in %). We consider that the observed feature is mostly unblended if the residual area is ≤25 %. A similar approach has been adopted in recent detections of complex organic molecules in line rich sources^38^. Note (a) indicates that the line is considered blended although the residual area is ≤25 %, because of a close (CH_2_OH)_2_ line. Values in parenthesis in the first column correspond to frequency uncertainties given in units of MHz.

## Data Availability

This Article makes use of data from projects 21A014 (Yebes 40 m), and 123-22 and 076-23 (IRAM 30 m). The observed spectra and LTE fits of the transitions of the different species presented in this work are available via Zenodo at https://doi.org/10.5281/zenodo.20081358 (ref. ^104^). The spectroscopic information of erythrulose can be found in ref. ^28^.

## Code availability

The MADCUBA software is publicly available at https://cab.inta-csic.es/madcuba/. A description of the package is provided in ref. ^85^. The Python-based script developed by ref. ^84^ is available via GitHub at https://github.com/andresmegias/gildas-class-python/. UCLCHEM can be downloaded freely from https://uclchem.github.io/. The KMC code is available on reasonable request to H.M.C.
