# Supplementary material for: Detection of a four-carbon sugar in interstellar space
Source: Nat Astron. 2026 Jul 13;10(9):1353–63. doi: 10.1038/s41550-026-02905-7 (PMC13581611; doi:10.1038/s41550-026-02905-7)
Supplement: Supplementary file 1 [file 41550_2026_2905_MOESM1_ESM.pdf]

nature astronomy

Supplementary information https://doi.org/10.1038/s41550-026-02905-7

Detection of a four-carbon sugar in interstellar space

In the format provided by the authors and unedited

Supplementary Information

LTE Analysis of aGg'-Ethylene Glycol [aGg'-($CH_2OH)_2$] with MADCUBA. As discussed in the main text, one of the possible precursors of erythrulose is ethylene glycol [or $(CH_2OH)_2$]. Ethylene glycol has several conformers, for which the aGg' conformer is the lowest in energy. Supplementary Figure 1 and Supplementary Table 1 report the spectra and the spectroscopic information of representative transitions of aGg'-$(CH_2OH)_2$. As seen from Supplementary Figure 1, aGg'-$(CH_2OH)_2$ is clearly detected with many clean (or slightly blended) bright transitions (peak intensities $\geq$30 mK). The physical parameters of aGg'-$(CH_2OH)_2$ derived using SLIM-AUTOFIT are reported in Table 1 of the main text.

Threshold to establish the level of line blending. As described in Methods, the criteria to establish whether a line is mostly-unblended or blended is that the residuals obtained after subtracting the contribution of the erythrulose LTE line fits to the observed spectra are $\leq$25%. To demonstrate the validity of this threshold, we have performed numerical simulations of mock spectra with flat line profiles (representing the most extreme case of heavy line blending) and with slightly-broader Gaussian line profiles with a linewidth = 1.3 $\times$ the original linewidth and a peak intensity = 1.1 $\times$ the original peak intensity (representing minor blending contribution; Supplementary Figure 2). The residuals are calculated by subtracting the Gaussian line (red shaded) area over a velocity range of $\pm$FWHM (or 2$\times$FWHM), covering 98.2% of the total area of the Gaussian profile. For the flat spectrum case (left panel in Supplementary Figure 2), the residuals amount to $\sim$50%, while for the slightly-blended case (right panel), they are $\leq$25%, respectively. This justifies our choice of $\leq$25% as the threshold for classifying lines as mostly-unblended.

Modeling of amorphous solid water (ASW) ice. Our mechanistic approach for the formation of erythrulose involves the non-diffusive association of activated glycolaldehyde (g) and ethylene glycol (e) on ASW ice. Non-diffusive chemistry has been invoked to explain the formation of COMs under cold ISM conditions, and it is especially important in sources where the temperature of the dust remains below 30 K[1, 2]. Therefore, the first step is to obtain the complex formed between both species (g-e) adsorbed on the ice surface. To simulate the ASW ice, we build a cluster of 100 water molecules starting by solvating an optimized water molecule with GFN2-xTB[3] through an Automated Molecular Cluster Growing calculation[4] implemented in the CREST[5] program at the same level of theory mentioned above. This cluster was optimized and used as the initial structure for performing molecular dynamics. The cluster of water molecules was encapsulated in a sphere of 27 Bohr radius to prevent evaporation of the water molecules, and a molecular dynamics simulation was carried out for 100 ps at 300 K using the GFN-FF force field.[6] The structure of the cluster was extracted every 10 ps and used as input for propagating another molecular dynamics simulation for 10 ps at a temperature of 10 K. These 10 clusters were optimized using the GFN2-xTB method, and their electronic energies were refined at the DFT level using the PW6B95(D3)[7] method in combination with the def2-TZVP basis set.[8] Among all these clusters, the one with the lowest relative energy compared to the least stable cluster was selected, in this case -100.0 kJ mol^{-1}.

Supplementary Table 1 Spectroscopic information of the aGg'-$(CH_2OH)_2$ transitions shown in Supplementary Figure 1.

Frequency (GHz)	Transition ($J', K_a', K_c', v' \rightarrow J'', K_a'', K_c'', v''$)	Log I (nm^2MHz)	E_{low} (cm^{-1})	Blending
84.439522	8,0,8,1→7,0,7,0	-4.690	9.3	clean
47.217410	4,0,4,1→3,0,3,0	-5.439	2.0	clean
74.232243	7,1,7,1→6,1,6,0	-4.921	7.1	clean
75.299876	7,0,7,1→6,0,6,0	-4.947	7.0	clean
92.975886	9,1,9,1→8,1,8,0	-4.600	11.9	clean
46.166540	5,1,4,0→4,1,3,1	-5.440	4.1	slightly blended (U-line)
49.581050	4,1,3,1→3,1,2,0	-5.502	2.5	slightly blended (CH_3C_3N)
83.624924	8,1,8,1→7,1,7,0	-4.857	9.4	clean
79.610634	9,0,9,0→8,0,8,1	-4.733	12.1	slightly blended ($CH_3{}^{13}CHO$)
102.689838	10,0,10,1→9,0,9,0	-4.442	14.7	slightly blended (Z-HCCCHNH & HNC_3)
45.547640	4,1,4,1→3,1,3,0	-5.605	2.3	clean
97.359028	9,2,8,1→8,2,7,0	-4.548	13.6	clean
86.655611	9,1,8,0→8,1,7,1	-4.634	13.3	blended (c-C_6H_5CN)
77.521974	7,2,6,1→6,2,5,0	-4.980	8.5	clean
80.892911	7,2,5,1→6,2,4,0	-4.957	8.6	clean
76.830836	8,1,7,0→7,1,6,1	-4.899	10.5	slightly blended (s-propanal)

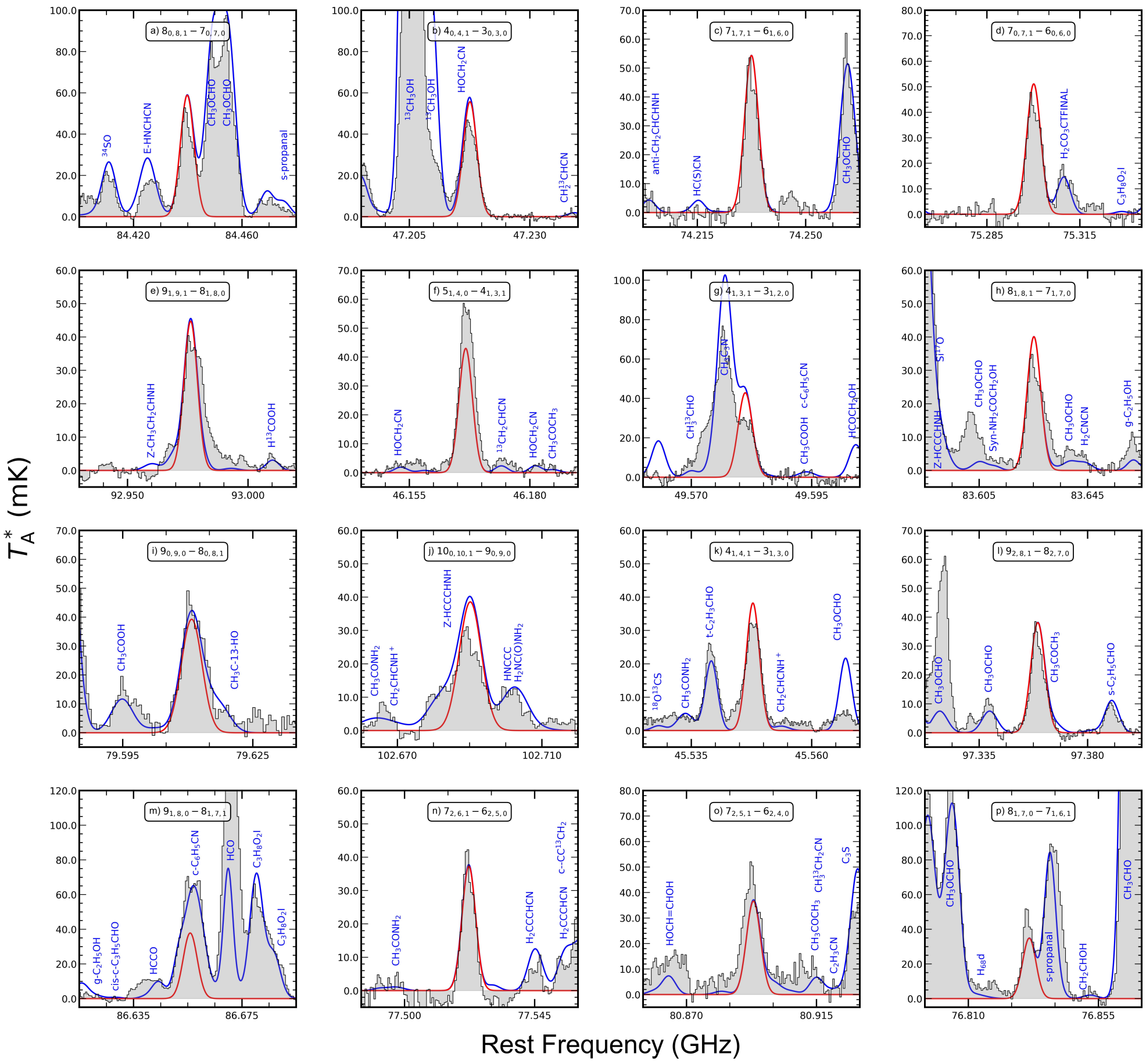

Supplementary Figure 1 Representative clean and slightly blended transitions of the lowest energy conformer of ethylene glycol, the aGg' conformer [aGg'-(CH_2OH)$_2$]. Red lines show the contribution of the individual aGg'-(CH_2OH)$_2$ lines, while the blue lines represent the predicted spectra after considering the emission from all molecular species identified toward G+0.693. The quantum numbers of the transitions are shown in the upper part of each panel. In blue, we also report other molecular species contributing to the total observed spectra in the vicinity of the aGg'-(CH_2OH)$_2$ lines.

Generation of the glycolaldehyde-ethylene glycol complex (g-e) in ASW. In our simulations, we assume that glycolaldehyde and ethylene glycol are formed close to each other on the ice surface. As glycolaldehyde and ethylene glycol are molecules with very polar groups, the aldehyde oxygen and the hydroxyl groups will point towards the water ice. With these premises, we placed several glycolaldehyde molecules around ethylene glycol with all polar groups pointing in the same direction. Each pair was optimized, resulting in only two possible structures: one structure in which the carbonyl group of glycolaldehyde interacts with a hydroxyl group of ethylene glycol (referred to as CO), and a second structure in which the two hydroxyl

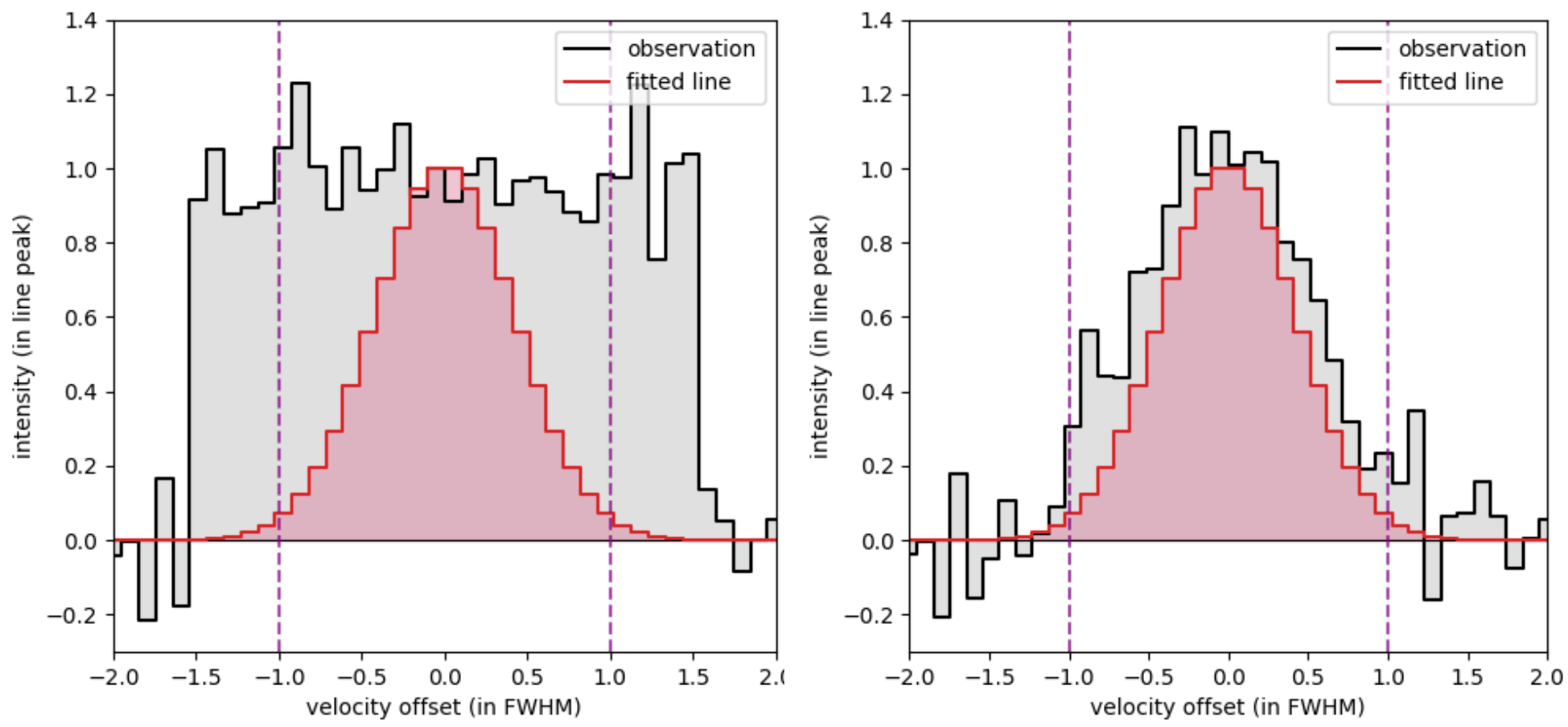

Supplementary Figure 2 Comparison between mock spectra (black histograms and grey shaded areas) and Gaussian line profiles (red histograms and red shaded areas). Left panel shows the most extreme case of blending with a mock flat spectrum, while the right panel shows the case of minor blending contribution. The noise in the mock spectra corresponds to 10% the peak value of the Gaussian line profile. Dashed vertical lines indicate the ±FWHM velocity range over which 98.2% of the area underneath the Gaussian profile is considered.

groups of the pair interact (OH). These two structures were optimized and used as fragments in a rigid docking study (aISS)[9], obtaining glycolaldehyde-ethylene glycol complexes (g-e) placed in different areas of the ice. For each complex, a sampling of 50 interactions was performed. All energies of the g-e complex on the ASW were refined at the aforementioned DFT level. The binding energies of the CO and OH complexes to the ASW (Supplementary Figure 3) were very similar (9349 ± 1162 cm^{-1} and 9157 ± 1279 cm^{-1}, respectively). However, the highest interaction for CO was 13264 cm^{-1}, and for OH, it was 11014 cm^{-1}. Then we selected the structure with the highest binding energy of the CO complex for the mechanistic study. Note that to calculate the binding energies, we used the energy of all fragments separately as a reference and are not ZPE-corrected because these attractive interactions are high in energy and the ZPE does not significantly alter the results.

Electronic structure calculations. All calculations carried out to study the reaction mechanism were done with the Gaussian 16 package[10]. For the association of the two fragments to occur in the ASW, first, the molecules must be activated, i.e. they must be radicals. The activation can occur after H abstraction reactions in both fragments. Hydrogen abstraction reactions are known to take place in ASW ice under interstellar conditions as demonstrated by laboratory experiments and astrochemical simulations[11–14]. The only hydrogens that can be abstracted in both fragments are those pointing out of the ASW ice, which correspond to C-H bonds

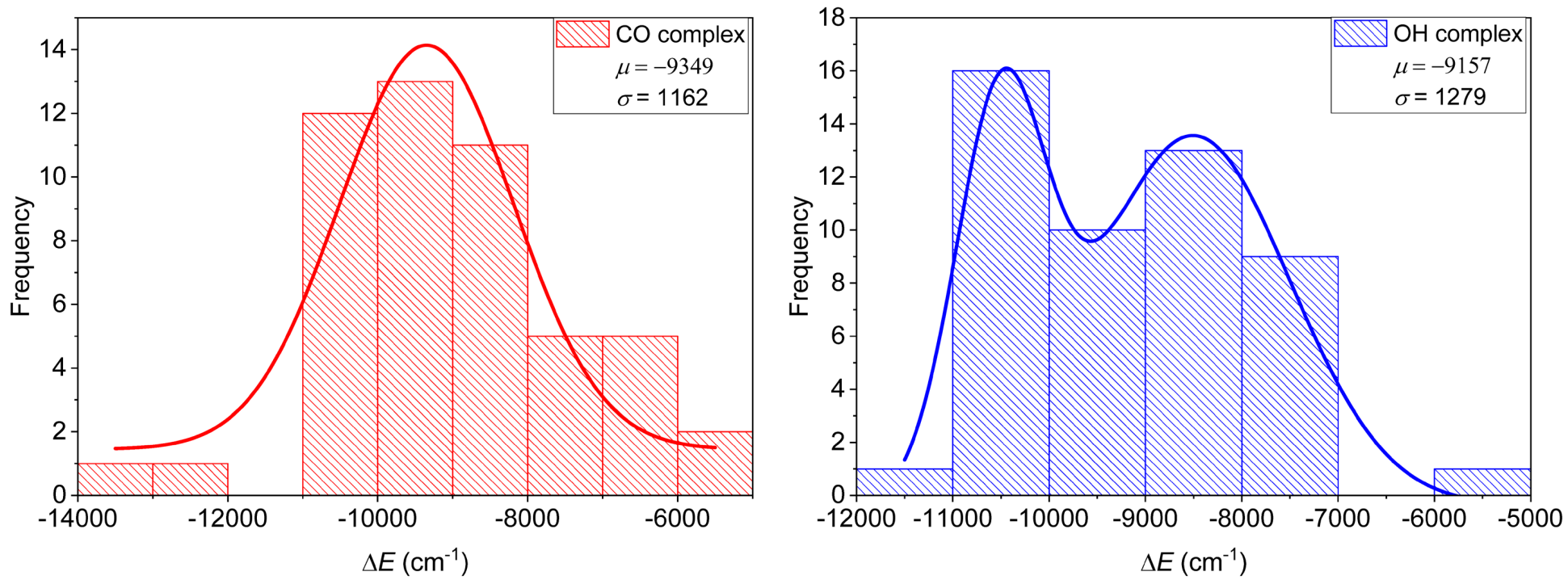

Supplementary Figure 3 Binding energies of the g-e CO (red) and OH (blue) complexes on the ASW. Solid lines represent gaussian fits to the binding energies.

(see optimized geometries in Figure 2). For this calculation, we used a three-layer ONIOM where the CO complex and the first solvation layer were treated at DFT level. The last water molecules included in the DFT level calculations interact with another layer of water molecules treated at a medium level of theory using the semiempirical PM7R6 method[15]. The rest of the water molecules, already away from the reaction area, were treated with the UFF force field[16], resulting in a PW6B95(D3)/def2-TZVP:PM7R6:UFF level of theory. All optimizations were carried out without restrictions, and the stationary points were characterized by frequency calculations at the same level of theory, showing no imaginary frequencies for energy minima and saddle points, respectively. For the calculation of the ZPE, a scale factor of 0.974 was used[17]. The harmonic approximation was used for frequency calculations, which introduces notable errors in entropy for normal modes of very low magnitudes. Therefore, all frequencies below 50 cm^{-1} were fixed at this value.

After the first H abstraction reaction, the complex adsorbed on the ice is a doublet (g*-e), so subsequent incident hydrogen atoms can approach the complex with the spin up or spin down, giving rise to two possible spin states, a singlet diradical and a triplet, respectively. The wavefunction for the high spin state was calculated and used as a reference for performing the calculation of the low spin state wavefunction (singlet diradical). In no case did we find transition states for the second H abstraction for the singlet diradical state. All transition states found for the second H abstraction are for triplet states.

Once the g*-e* complex is formed, which is in the triplet state, it must change the spin of one of the carbons to form erythrulose. Therefore, the singlet-triplet ISC transition was considered. The spin-orbit coupling was calculated at the complete active self-consistent field[18] with an active space of six electrons in five molecular orbitals, leaving a CASSCF(6e,5o)/def2-TZVP level of theory. These calculations were carried out with the ORCA software[19]. To do this, we removed all the water molecules treated with the UFF force field and the ones that were treated with the PM7R6 method were calculated with the GFN2-xTB.

Kinetic calculations. Since all polar groups in the g-e complex are oriented towards the ice surface, we focused on the abstraction of hydrogen atoms that remain non-interacting with the surface. To obtain the relative energies of the transition states, an Intrinsic Reaction Coordinate (IRC) calculation was performed to obtain the geometry of the reactant complex, which was taken as a reference to obtain the energy barriers and reaction energies. The rate constants were obtained using transition state theory (TST) using the Pilgrim software[20]:

$$k(T) = \kappa \frac{k_B T}{h} \frac{Q^{\text{SP}}_{\text{rot}} Q^{\text{SP}}_{\text{vib}} Q^{\text{SP}}_{\text{elec}}}{Q^{\text{C}}_{\text{rot}} Q^{\text{C}}_{\text{vib}} Q^{\text{C}}_{\text{elec}}} e^{\frac{-V^{\text{SP}}}{k_B T}} \tag{1}$$

where k_B and h are the Boltzmann and Plank constants, respectively; Q refers to the partition function with the super index C and SP corresponding to the complex and saddle point, respectively; and V^{SP} is the potential energy of the saddle point. Since we are computing a mechanism on a surface, the rotational partition functions $Q^S_{rot}P$ and Q^C_{rot} were set to 1. The rotational partition function was not fixed for molecular hydrogen since the relative energy of the products with respect to the complex was obtained with respect to the energy asymptote of the exit channel. Else, the rotational partition function was set to unity for the newly formed radical, as was done for the complex and the saddle point. The classical rate constants obtained with TST were corrected by multiplying them by the tunneling transmission coefficient (κ) using the Eckart approximation[21]. The final step of the reaction, in which the singlet diradical recombines to form erythrulose, follows a Langmuir–Hinshelwood mechanism for which we could not calculate rate constants since we could not characterize any transition structure connecting the two minima. To approximate the ISC rate in the diradical intermediate g*-e* we use Marcus' semi-classical theory, given by[22, 23]

$$k_{\text{ISC}}(T) = \frac{2\pi}{h} V^2_{\text{SOC}} \sqrt{\frac{\pi}{\lambda k_B T}} e^{\frac{-(\lambda+\Delta G)^2}{4\lambda k_B T}} \tag{2}$$

where ΔG is the free energy difference between the two spin states for their equilibrium geometries, λ is the reorganization energy, which was calculated following the four-point technique proposed by Nelsen[24] and V_{SOC} is the spin-orbit coupling between the two spin states.

Further details on the reaction mechanism between glycolaldehyde and ethylene glycol on ASW. In the main text we conclude that reaction 3-g-e is the most favourable reaction on ASW because its energy barrier (4.1 kJ mol^{-1}) is significantly lower than those of the H abstraction reactions 1-g-e, 4-g-e, 5-g-e or 2-g-e (see panel **a** in Figure 2). Whereas reactions 1-g-e, 4-g-e and 5-g-e present, respectively, energy barriers of 11.5, 22.2 and 14.9 kJ mol^{-1}, the H addition to the C atom in reaction 2-g-e is 23.4 kJ mol^{-1}. Therefore, all these reactions cannot compete with reaction 3-g-e based on their classical energy barriers. Although reaction 1-g-e has a classical energy barrier just 7.4 kJ mol^{-1} above that of the reaction 3-g-e, the rate constant is significantly lower because the imaginary frequency of the saddle point is 289.4 cm^{-1} lower than that found for reaction 3-g-e, which leads to a very significant decrease in the transmission coefficient (see below).

When the activated complex g*-e is formed after the initial H abstraction reaction 3-g-e (see Figure 2, panel **a**, in the main text), it adopts a doublet spin state. Subsequent collisions with additional H atoms can then result in the formation of two possible spin states. When the system is in a triplet state, the approach of H towards the activated carbon is repulsive, while collisions with the available H atoms from g*-e lead to further H abstractions, forming H_2 and a doubly activated complex in a triplet state (g*-e*). We note that H atoms with antiparallel spin approaching the activated carbon of the aldehyde could revert to the inactivated g-e complex, as the potential between these two atoms is attractive and barrierless. This reversibility to the g-e complex could hinder the reaction process or lead to the desorption of the reactants from the ice, given that hydrogenation of the activated aldehyde carbon is exothermic by 356.8 kJ mol^{-1}. Nevertheless, it is well known that collisions with atomic hydrogen on molecules within ice tend towards more complex systems through diradical recombination. This is due to the different orientation of the new incident H atoms in subsequent collisions added to the orientation of the COMs on the ASW, which allows the reaction to proceed through more H abstraction reactions[1, 2]. In our case, the incoming H atom towards hydrogen (3) of ethylene glycol in the g*-e complex (Figure 2, panel **b**) has a different orientation from that required to collide with the activated carbon of g*-e, yielding the g*-e* complex shown in Figure 2 (panel **c**) in an extremely fast process.

As shown in Figure 2 (panel **b**), there are three highlighted hydrogen atoms in the g*-e complex available to react with colliding H when the system is in the triplet state. However, our calculations show that the formation of the pre-reactive complex for the abstraction reaction 1-g*-e is repulsive, being 3.0 kJ mol^{-1} above the reactants' asymptote. In contrast, the pre-reactive complex for the reaction 3-g*-e is 25.0 kJ mol^{-1} below the reactants' asymptote. Therefore, the energy difference between these two complexes is 28.0 kJ mol^{-1}, which makes reaction 1-g*-e unfeasible under ISM conditions. The inspection of both complexes shows that the interaction energy of g*-e with H at two different positions is not the reason for this large energy difference. The geometries of the pre-reactive complexes for hydrogen (1) and hydrogen (3) without considering the incoming hydrogen are also very similar, showing a geometry RMSD deviation of 1.5 Å. The main difference between the two geometries lies in the orientation of ethylene glycol on the ASW ice, which slightly rotates its position along the C-C bond (see Supplementary Figure 4). This small reorientation induces a change in the O-C-C-O dihedral angle, being 58.8° and 65.5° for the least stable and the most stable complex, respectively. From an electronic perspective, the greater the distance between the oxygen atoms of these two hydroxyl groups, the lower the steric repulsion. The change in orientation decreases the directionality of the hydrogen bond with respect to the carbonyl group, which destabilizes the system.

The H abstraction reaction 2-g*-e forms hydroxyketene through a hydroxyketene-ethylene glycol complex, while reaction 3-g*-e yields the doubly activated complex g*-e*. These two reactions show almost identical rate constants, with the 3-g*-e reaction being slightly favorable as can be seen from the branching ratios (Figure 2, panel **g**). The energy barriers for the reactions 2-g*-e and 3-g*-e are 12.7 and 15.6 kJ mol^{-1} and the reaction energies are -111.5 and -36.2 kJ mol^{-1} respectively. Although the classical

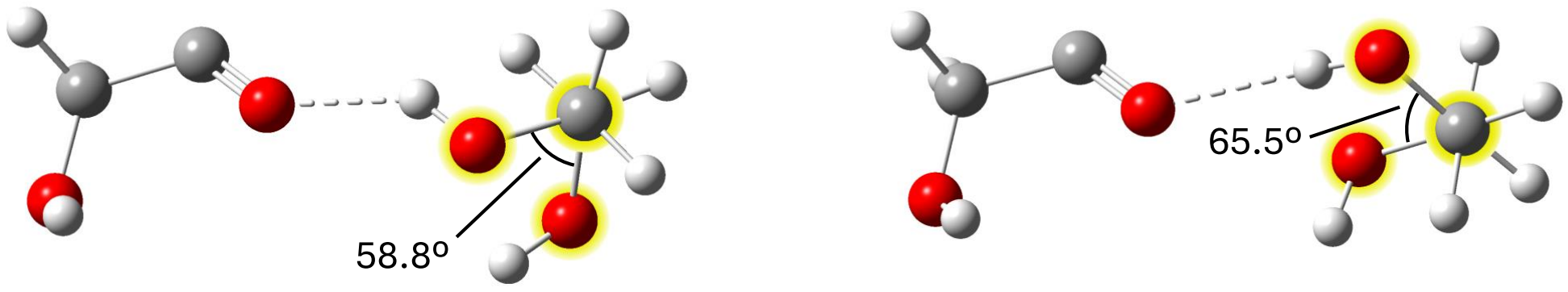

Pre-reactive complex for
1-**g*-e**

Pre-reactive complex for
3-**g*-e**

Supplementary Figure 4 Optimized geometries of the pre-reactive complexes for reactions 1-g*-e and 3-g*-e. For the shake of clarity, water molecules and the H atom have been removed.

energy barrier favors the reaction 2-g*-e, the quantum corrected rate constants are slightly higher for 3-g*-e since the transmission coefficients (κ) are higher for the latter. It can be noticed qualitatively by inspecting the imaginary frequency of the saddle points of reactions 2-g*-e and 3-g*-e, which are 1018.5i and 1235.3i cm^{-1}, respectively.

Once the g*-e* complex is formed in the triplet state, one of the electron spin must change to form erythrulose. The ISC process depends mainly on three factors: the spin-orbit coupling between the two states (VSOC), the change in geometry (reorganization energy, λ) and the free energy difference between the singlet and triplet states. The spin-orbit coupling between these two states is very low (0.002 cm^{-1}), because there is no change in the orbital angular momentum after the spin changes, which makes these two states poorly electronically coupled. The change in the geometry of the singlet and triplet states is practically negligible, leading to a near-zero rearrangement energy (2.1×10^{-4} eV), which greatly increases the transition probability. This, coupled with the fact that both states are nearly degenerate near the absolute zero, makes the spin change very fast at low temperatures (Figure 2, panel **h**). Although this process is fast, it is still slower than the two previous abstraction reactions. We note that the ISC process does not occur for the ketene-ethylene glycol complex. After the abstraction reaction 2-g*-e, a ketene is formed in the triplet state, where the C-C-O angle is 130°, whereas the singlet state shows a C-C-O angle of 177.2°. This geometrical change induces a considerable increase of λ (2.8 eV), which reduces notably the probability of transition. Moreover, the free energy difference between the two states is in the range 114.5-154.6 kJ mol^{-1} for T=10–300 K, making the transition non-viable. In this case, the spin-orbit coupling is higher than for the g*-e* complex, being 3.22 cm^{-1}.

Once the doubly activated g*-e* complex is in the singlet state, recombination of the two fragments produces erythrulose. In many cases, this recombination occurs by a barrierless reaction after the second H abstraction reaction[1, 2]. However, we succeeded in characterizing the singlet diradical as an energy minimum. All attempts to characterize a saddle point leading directly to erythrulose were unfruitful, pointing to the fact that if any transition state existed between these two minima (g*-e* and erythrulose) it would be located in a very flat surface. After performing a relaxed scan by varying the C-C coordinate, we were able to detect a small energy maximum at a C-C distance of 3.4 Å of 1.1 kJ mol^{-1} above g*-e* (Supplementary Figure 5). Such

an electronic energy barrier would imply a k(T) of the order of 10^8 s^{-1}. The release of the C-C degree of freedom would give rise to a more relaxed structure, decreasing the electronic energy of the system; the inclusion of ZPE would yield a lower barrier; and the tunneling effect is probably not negligible even for a wide barrier at 20 K; therefore, we can qualitatively describe this process as an extremely rapid reaction that will hardly influence the overall rate of the mechanism. Overall, the rate-determining step of the whole process is the intersystem crossing (ISC) rate of g*-e*.

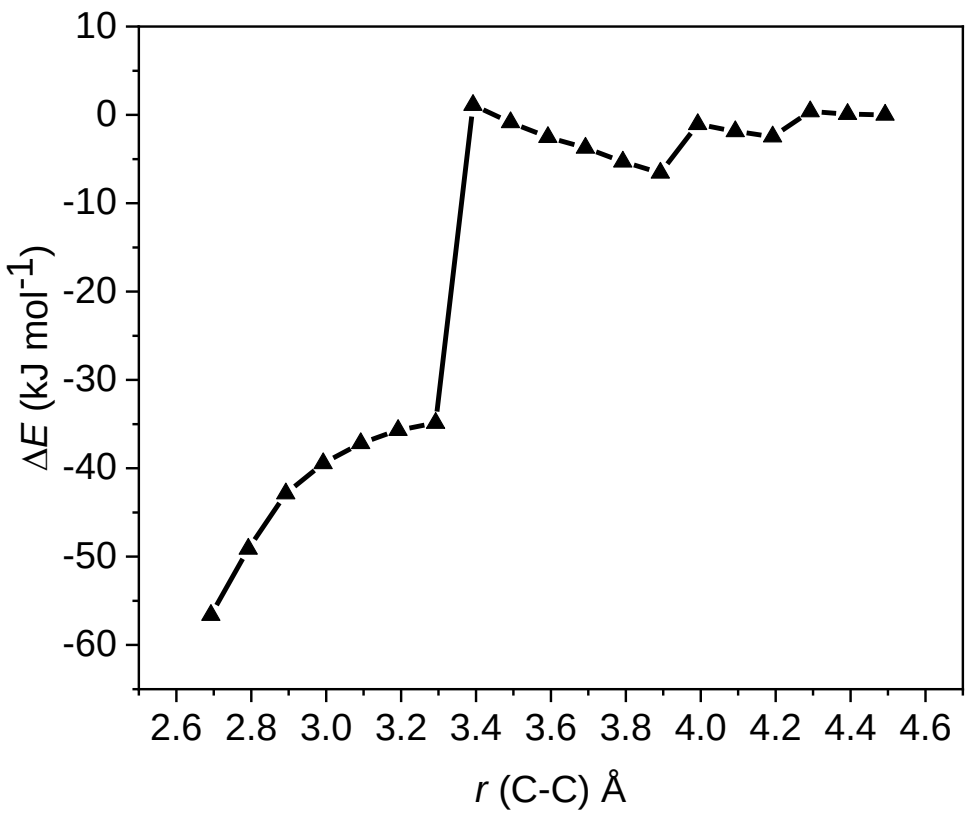

Supplementary Figure 5 Relaxed scan along the C-C coordinate of the g*-e* complex. Electronic energy change relative to the doubly activated g*-e* complex in the singlet state. The degree of freedom scanned is the C-C coordinate which corresponds to the two atoms holding the unpaired electrons. For each optimization along the C-C coordinate no constraints were imposed to any degree of freedom.

CASSCF active space for the g*-e* and ketene-ethylene glycol complexes. The multiconfigurational calculations of the g*-e* and ketene-ethylene glycol complexes were carried out to derive the spin-orbit coupling. The molecular orbitals included in the active space in both complexes are the π and π^* orbital pairs, the lowest energy lone pair of the oxygen atom (n) as well as the p type orbitals bearing the unpaired electrons (Supplementary Figure 6). In the g*-e* complex, four out of five orbitals belong to the glycolaldehyde radical, that is, the π_z^* and π_z of the C-O bond, the lone pair of the oxygen atom (n_x) and the p_x orbital bearing the unpaired electron. The fifth orbital of the g*-e* complex is the p_y orbital bearing the unpaired electron of the ethyleneglycol moiety.

The five molecular orbitals of the selected active space in the ketene-ethylene glycol complexes are in the ketene moiety. The orbitals are: The π_z of the C-C bond, which bear one of the unpaired electrons, its π_z^* pair, the π_z of the C-O bond; the doubly occupied n_x of the oxygen atom and the p_x orbital bearing the other unpaired electron.

Summary of the reaction mechanism. A summary of the formation mechanism of erythrulose on ASW ice from glycolaldehyde and ethylene glycol is given in Supplementary Figure 7. In Supplementary Table 2 we provide the rate constants of the

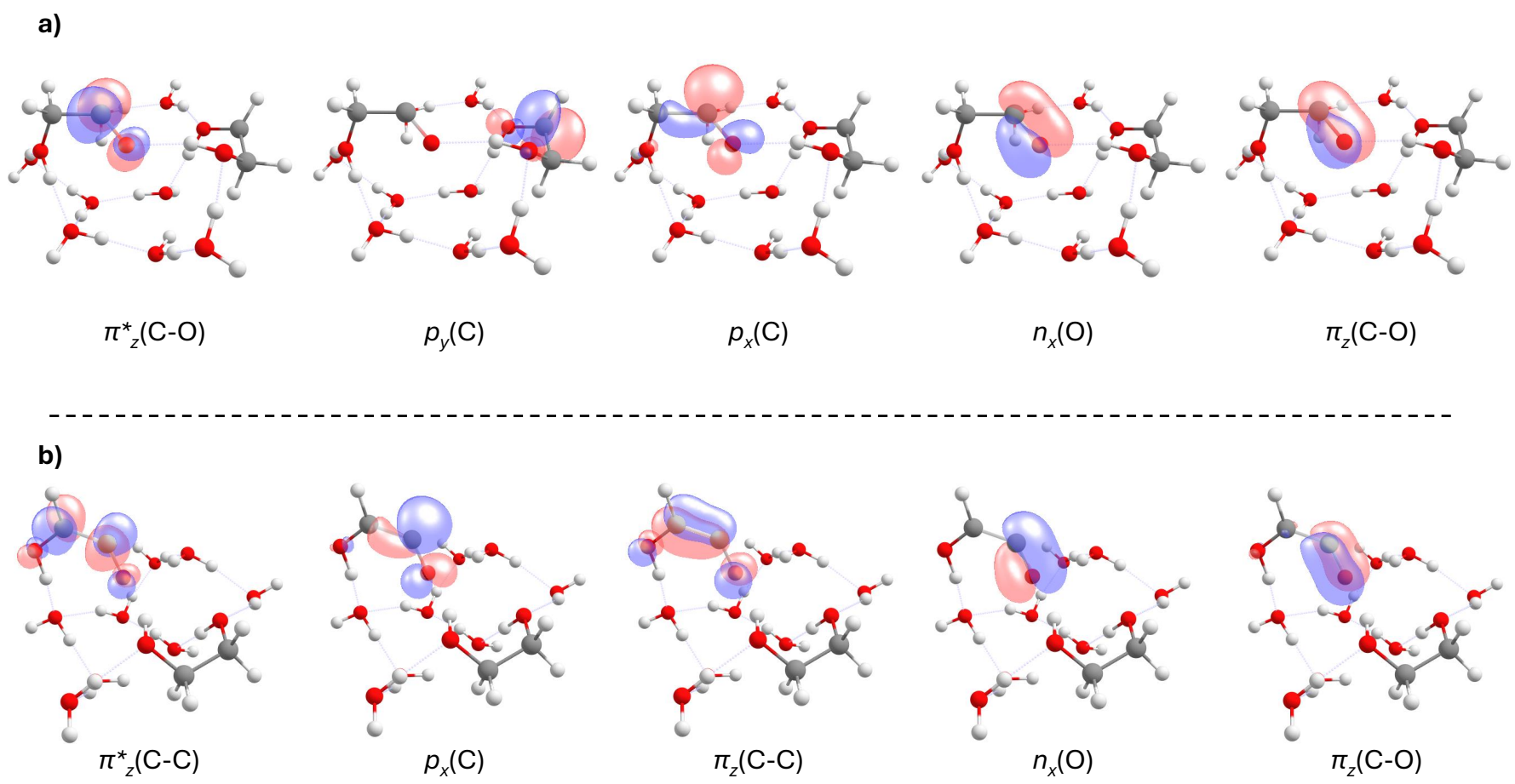

Supplementary Figure 6 Active orbitals of the active space for the CASSCF(6e,5o)/def2-TZVP calculations. Panel a) shows the active orbitals for the g*-e* complex, while panel b) for the ketene-ethylene glycol complex. Some water molecules have been removed in order to enhance the clarity of the images.

individual H abstraction reactions and of the radical-radical recombination reaction (the ISC process) reported in Figure 2 and described in the main text.

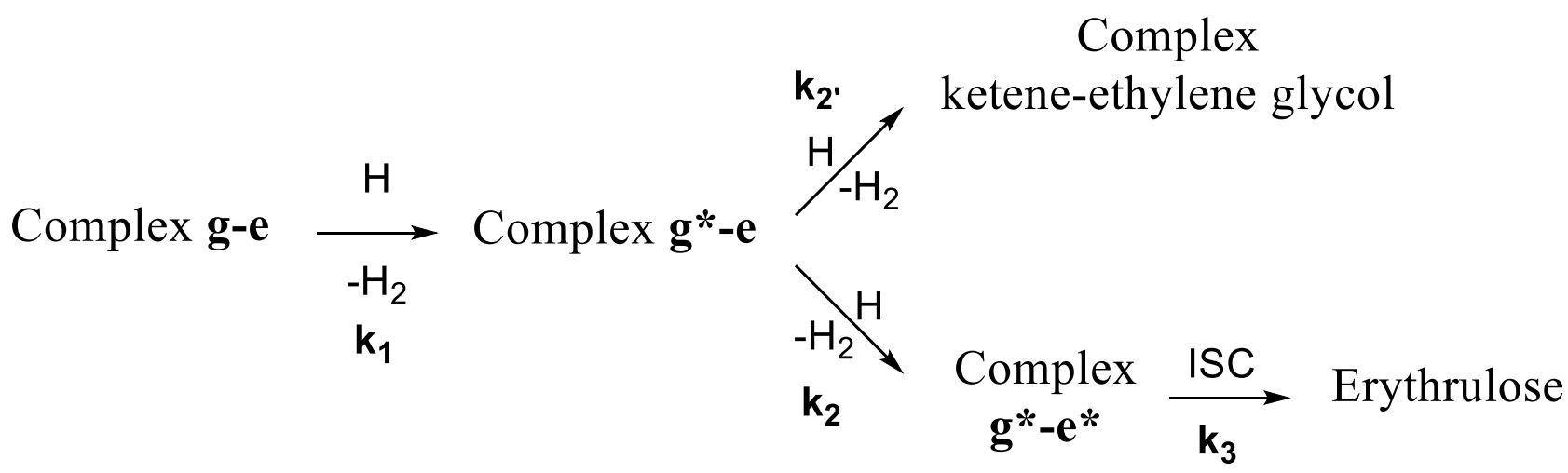

Supplementary Figure 7 Summary of the mechanism of formation of erythrulose on ASW from glycolaldehyde (g) and ethylene glycol (e). Reaction 3-g-e in Figure 2 is governed by rate constant k_1. Reactions 2-g*-e and 3-g*-e in Figure 2 present, respectively, rate constants k_2' and k_2. And the ISC reaction of complex g*-e* in Figure 2 finally yields erythrulose at a rate constant k_3. The values of these rate constants are given in Supplementary Table 2 for a temperature range between 20-300 K.

Supplementary Table 2 Quantum tunneling-corrected rate constants of reactions 3-g-e (k_1), 2-g*-e (k_2') and 3-g*-e (k_2), and of the ISC reaction of the g*-e* complex (k_3).

T (K)	k_1 (s^{-1})	k_2' (s^{-1})	k_2 (s^{-1})	k_3 (s^{-1})
20	3.08E+10	9.00E+06	9.72E+06	2.44E+05
30	6.55E+10	1.80E+07	2.01E+07	1.04E+05
40	1.13E+11	2.79E+07	3.22E+07	3.99E+04
50	1.71E+11	3.85E+07	4.53E+07	1.47E+04
60	2.35E+11	5.04E+07	5.93E+07	5.31E+03
70	3.02E+11	6.42E+07	7.48E+07	1.91E+03
80	3.69E+11	8.07E+07	9.18E+07	6.82E+02
90	4.31E+11	1.01E+08	1.11E+08	2.24E+02
100	4.91E+11	1.26E+08	1.33E+08	8.01E+01
120	5.98E+11	1.94E+08	1.88E+08	1.02E+01
140	6.93E+11	2.96E+08	2.66E+08	1.31E+00
160	7.77E+11	4.33E+08	3.72E+08	1.69E-01
180	8.49E+11	6.16E+08	5.15E+08	2.39E-02
200	9.15E+11	8.42E+08	7.06E+08	3.10E-03
220	9.73E+11	1.10E+09	9.41E+08	4.86E-04
240	1.02E+12	1.40E+09	1.24E+09	5.26E-05
260	1.07E+12	1.72E+09	1.60E+09	8.30E-06
280	1.12E+12	2.06E+09	2.01E+09	1.09E-06
300	1.15E+12	2.40E+09	2.48E+09	1.57E-07

References

[1] Lamberts, T., Markmeyer, M. N., Kolb, F. J. & Kästner, J. Formation of acetaldehyde on co-rich ices. *ACS Earth and Space Chemistry* **3**, 958–963 (2019).

[2] Simons, M. A. J., Lamberts, T. & Cuppen, H. M. Formation of coms through co hydrogenation on interstellar grains. *Astron. Astrophys.* **634**, A52 (2020).

[3] Bannwarth, C., Ehlert, S. & Grimme, S. Gfn2-xtb—an accurate and broadly parametrized self-consistent tight-binding quantum chemical method with multipole electrostatics and density-dependent dispersion contributions. *Journal of Chemical Theory and Computation* **15**, 1652–1671 (2019).

[4] Spicher, S., Plett, C., Pracht, P., Hansen, A. & Grimme, S. Automated molecular cluster growing for explicit solvation by efficient force field and tight binding methods. *Journal of Chemical Theory and Computation* **18**, 3174–3189 (2022).

[5] Pracht, P., Bohle, F. & Grimme, S. Automated exploration of the low-energy chemical space with fast quantum chemical methods. *Phys. Chem. Chem. Phys.* **22**, 7169–7192 (2020).

[6] Spicher, S. & Grimme, S. Robust atomistic modeling of materials, organometallic, and biochemical systems. *Angewandte Chemie International Edition* **59**, 15665–15673 (2020).

[7] Zhao, Y. & Truhlar, D. G. Design of density functionals that are broadly accurate for thermochemistry, thermochemical kinetics, and nonbonded interactions. *The Journal of Physical Chemistry A* **109**, 5656–5667 (2005).

[8] Weigend, F. & Ahlrichs, R. Balanced basis sets of split valence, triple zeta valence and quadruple zeta valence quality for h to rn: Design and assessment of accuracy. *Phys. Chem. Chem. Phys.* **7**, 3297–3305 (2005).

[9] Plett, C. & Grimme, S. Automated and efficient generation of general molecular aggregate structures. *Angewandte Chemie International Edition* **62**, e202214477 (2023).

[10] Frisch, M. J. *et al. Gaussian 16 Revision A.03* (2016).

[11] Fedoseev, G. *et al.* Formation of Glycerol through Hydrogenation of CO Ice under Prestellar Core Conditions. *Astrophys. J.* **842**, 52 (2017).

[12] Ioppolo, S. *et al.* A non-energetic mechanism for glycine formation in the interstellar medium. *Nature Astronomy* **5**, 197–205 (2021).

[13] Garrod, R. T. *et al.* Formation of Complex Organic Molecules in Hot Molecular Cores through Nondiffusive Grain-surface and Ice-mantle Chemistry. *Astrophys. J. Supp. Series* **259**, 1 (2022).

[14] Borshcheva, K. *et al.* Formation of Complex Organic Molecules in Prestellar Cores: The Role of Non-Diffusive Grain Chemistry. *Astrophys. J.* **990**, 163–188 (2025).

[15] Stewart, J. J. P. Optimization of parameters for semiempirical methods vi: more modifications to the nddo approximations and re-optimization of parameters. *Journal of Molecular Modeling* **19**, 1–32 (2013).

[16] Rappe, A. K., Casewit, C. J., Colwell, K. S., Goddard, W. A. I. & Skiff, W. M. Uff, a full periodic table force field for molecular mechanics and molecular dynamics simulations. *Journal of the American Chemical Society* **114**, 10024–10035 (1992).

[17] Alecu, I. M., Zheng, J., Zhao, Y. & Truhlar, D. G. Computational thermochemistry: Scale factor databases and scale factors for vibrational frequencies obtained from electronic model chemistries. *Journal of Chemical Theory and Computation* **6**, 2872–2887 (2010).

[18] Roos, B. O., Taylor, P. R. & Sigbahn, P. E. A complete active space scf method (casscf) using a density matrix formulated super-ci approach. *Chemical Physics* **48**, 157–173 (1980).

[19] Neese, F., Wennmohs, F., Becker, U. & Riplinger, C. The orca quantum chemistry program package. *JChPh* **152**, 224108 (2020).

[20] Ferro-Costas, D., Truhlar, D. G. & Fernández-Ramos , A. *Pilgrim-version 2020.2 (University of Minneapolis, Minnesota, MN, and Universidade de Santiago de Compostela, Spain)* (2020).

[21] Eckart, C. The penetration of a potential barrier by electrons. *PhRv* **35**, 1303 (1930).

[22] Marcus, R. A. Chemical and electrochemical electron-transfer theory. *Annual Review of Physical Chemistry* **15**, 155–196 (1964).

[23] Marcus, R. & Sutin, N. Electron transfers in chemistry and biology. *Biochimica et Biophysica Acta (BBA) - Reviews on Bioenergetics* **811**, 265–322 (1985).

[24] Nelsen, S. F., Blackstock, S. C. & Kim, Y. Estimation of inner shell marcus terms for amino nitrogen compounds by molecular orbital calculations. *Journal of the American Chemical Society* **109**, 677–682 (1987).
