# Supplementary material for: Detection of a four-carbon sugar in interstellar space
Source: Nat Astron. 2026 Jul 13;10(9):1353–63. doi: 10.1038/s41550-026-02905-7 (PMC13581611; doi:10.1038/s41550-026-02905-7)
Supplement: Supplementary file 2 [file 41550_2026_2905_MOESM2_ESM.pdf]

nature portfolio

Peer Review File

Detection of a chiral four-carbon sugar in interstellar space

Corresponding Author: Dr Izaskun Jimenez-Serra

This file contains all reviewer reports in order by version, followed by all author rebuttals in order by version.

Version 0:

Reviewer comments:

Reviewer #1

(Comments for the Author)
The features highlighted in Figure 1 are very compelling for the unique detection of this sugar, the first interstellar sugar. Hence, this journal is a fitting venue for this exciting paper. The supporting information from the quantum chemical computations on the surfaces as well as the kinetics modeling give a clear indication that this molecule should be able to be formed under the conditions of the observed region. Hence, the discovery has both firm and supportive evidence. The manuscript is well constructed, and there were only a few items that I thought the authors could or should address.

The last paragraph of the "Introduction" is actually results and should be moved down a section. This leaves a thin introduction, but one that should be able to stand on its own.

The authors really need to cite some papers by other groups. For instance, the ice grain work producing sugars led by Kaiser should also be cited (DOI: 10.1126/sciadv.adl323 & DOI: 10.1073/pnas.2320215121). Additionally, a couple of citations to recent detections by the group led by Cernicharo are warranted such as the detection of trans-propenethial.

Several reference calls go to superscripts that should be on the line of the text.

The computational methods section has a discussion about the positions of the molecules. The text should point to Figure 2 to make this more concrete.

Reviewer #2

(Comments for the Author)
Overall, the authors are making a bold new claim presenting evidence for the first detection of an actual sugar - Erythrulose - detected in the gas phase toward the high mass molecular cloud G+0.693.

The data the authors have collected on this source is remarkable - covering frequencies from about 35 - 150 GHz and the chemistry in this source is definitely unique as it contains molecular species in the gas phase that are not detected in any other astronomical environment including NH2OH and unique S-bearing molecules such as HCOSH and CH3SCH3. The explanation for this unique molecular complexity is attributed to wide-spread shocks pervasive in the region that leads to the sputtering of unique chemical species off grain surfaces so they can be detectable in the gas-phase.

The observing team over the course of many years has provided evidence for some of the most complex astronomical molecules ever detected - surpassing even the molecular complexity found in Sgr B2N. And in this work, they again present evidence for one of the largest organic astronomical molecules ever claimed which would have profound implications on understanding the nature and formation of sugars, and possibly even amino acids, under interstellar conditions. If shown to be correct, it would be one of the most significant contributions to the field of astrochemistry and deserve publication in Nature Astronomy. Unfortunately, after critical review of the data presented, there is just not enough evidence present to make such an revolutionary claim. I strongly recommend the authors continue on this path started in this work and gather additional evidence in support of this detection.

Major concerns:
The data presented in Figure 1 of the proposed 27 transitions do not show any clear detection of even 1 individual transition

that can be defined as "clean" by either the criteria references by the authors themselves or from previous studies (Snyder et al. 2005 & Xue et al. 2019 - the accepted standard in the field for what can be considered an unblended transition). The first identification of an actual interstellar sugar - glycolaldehyde has often been referred to as a sugar but definitely is not by formal chemical definitions - would be remarkable. As such, a remarkable claim requires remarkable evidence. That remarkable evidence is not presented in these data. Especially when you compare the data presented in Fig 1 with the supplemental data presented on ethylene glycol where multiple, well defined and clean transitions are present in the data.

In addition, there are several molecules that are labeled in the spectrum as possible interloper species - looking at just the top row of figure 1, there are supposed interlopers of CH3C6H, HCCCH2CN, HC9N and c-C6H5CN. These species have largely been found in cold dark clouds and while the conditions presented in G+0.693 may be similar to a dark cloud environment, such large hydrocarbons are not typically found in regions as massive and complex as G+0.693.

Conversely, there is NO evidence for the transition of CH3COOH around 45.010 GHz - which I would assume to be present because there is both a label and modeled spectral feature… yet the spectrum at that frequency is complete blank showing no evidence of acetic acid.

Overall, the proposed identification a new sugar species is called into question given the lack of ANY clear, clean and unblended transitions as well as the questionable identification of transitions of other molecular species that are 1) clearly not represented at all in the spectrum presented and 2) would be the first identification of a species in a region that is not a cold, dark cloud (or in this region with considerably less uv-flux and where shocks are pervasive.).

It is good that the authors present the spectroscopic data in the supplemental material but the full spectroscopic catalog would also be necessary to include so that a full analysis can be done by the reader. The authors also make the claim that no other transitions are missing from any of the other data in their survey as they either are severely blended and/or lie below the measured noise level. Presenting even some of these additional transitions/data to the reader would provide a better case for the data presented in figure 1 as "clean" transitions. My assumption, without actually seeing any of these additional data, is that the blended transitions throughout the 2 and 3mm bands would not look any worse than the data presented in Figure 1 - which again is fueling my skepticism that there are any clear transitions of this molecule present in these data - and even if there was 1 completely clean transition, would that be enough to make the claim of the detection of a new sugar molecule? That is doubtful as a region that is as complex as G+0.693 would contain hundreds (if not thousands) of U-lines that may be attributed to other well-known, yet uncharacterized species (e.g. isotopologues, higher vibrational states). Finally, it should be stressed again that the presentation of a rotation diagram provides not additional evidence for a claimed new detection and should not be used in support of a new detection.

Finally, the modeling work done looks complete and extensive. The authors have done an amazing amount of work trying to fundamentally explain the possible formation of a 4-C sugar in this environment. However, it is beyond my area of expertise to comment on the actual chemical formation routes to the proposed formation routes to 3- and 4-carbon sugars. What I can comment on is the calculated relative abundance ratios between the claimed detected molecules with respect to the model predictions. If I am reading figure 3 correctly, the abundance of Erythrulose is not orders of magnitude larger than the 3-C sugars… at any time or in any model. Yet, the observed abundances are (at least) 2 orders of magnitude different. The authors make several assumptions as to why the 3- and 4-C sugars may be different in abundance given things like the 3-C sugars are more readily destroyed in the gas-phase and that both species may be harder to get off the grain surface or rapidly re-absorb onto the surface.

The issue however is clear, there is no fundamental reason given, from a chemical kinetics perspective, as to why this particular 4-C sugar should be preferentially formed with respect to other chemical similar (and simpler) species. Which again, really calls into question as to whether the species is there at all in the data at the claimed abundance. Given the formation models present in this work, the 3-C sugars should absolutely be present under these conditions - may be a factor of 2 lower in abundance at the most but the lack of any evidence for the simpler, smaller species without any good evidence or explanation, again, calls into question the detection of one of the largest, and most complex sugar ever claimed.

Reviewer #3

(Comments for the Author)
The manuscript NATASTRON-25091222, "Sugar in interstellar space" by Jimenez-Serra et al. reports the interstellar detection of erythrulose, a four-carbon ketose sugar, in the molecular cloud G+0.693-0.027. No other sugar has yet been detected in the ISM, despite many sugars being found in the carbonaceous material in meteorites. This result is of great potential interest in the fields of astrochemistry and astrobiology, as sugars are essential biomolecules and their formation on early Earth is poorly understood. The detection of this particular sugar is also surprising, as searches for smaller three-carbon sugars glyceraldehyde and dihydroxyacetone in this and other sources have not previously led to detections.

The authors showcase their results with a fair and balanced presentation of the difficulties in detecting such a molecule in a dense spectrum containing many lines from numerous interstellar species. They then go through a detailed analysis of possible formation mechanisms to explain the unexpectedly high fractional abundance of this molecule relative to other related species previously detected in this source.

Despite many positive aspects of the manuscript, I have serious reservations about the interpretation of the observational results. Examining Figure 1 in detail reveals that most of the brightest erythrulose lines that are reported herein are actually

significantly blended with lines arising from other known molecules. The lines that are not blended are considerably weaker than the others. And yet the authors claim that there are unblended lines of significant intensity -- where? I do not see them in Figure 1. Confused by the author's claim that "[f]ourteen out of 27 transitions are either clean or slightly blended with other molecular species" I turned to Extended Data Table 1. And this is where I found the source of my confusion. Most of the features listed here as "clean" are actually blended features in the observational spectra. The distinction is that they are blended with features that have not yet been identified. An example is the feature at 40.93 GHz, which is listed in this table as clean and shown in Figure 1 as the third panel from the left in the top row. While indeed this feature is clean from other *known* lines, the modeled spectrum in red only accounts for about half of the integrated intensity in the observed feature. The rest of the intensity must be arising from a different molecule that has not yet been identified. There are many such features shown in Figure 1. Simply put, these are not clean lines -- these are blended lines. Labeling them as clean leads to confusion at best and misinterpretation of the the results at worst. This calls into question the validity of the detection. And that calls into question the necessity for the rest of the study. If this detection is not valid, then the molecular abundance derived is not reliable, and the rest of the analysis and interpretation is based on incomplete or erroneous information. The authors need to better justify their claim of a detection before the rest of the manuscript can be considered.

If one proceeds through the rest of the manuscript based on the assumption that the detection is valid, the other steps of the analysis and interpretation are well done. Additional comments that could further enhance the rest of the manuscript include:

1. The statement "Given that glyceraldehyde and dihydroxyacetone cannot serve as precursor molecules for erythrulose, we turn our attention to smaller building blocks of this C4 sugar." needs to be backed up with more information and references. Why can these two smaller sugars not serve as precursors to erythrulose?

2. In the rotation diagram analysis presented on page 7, the authors did not make clear whether the simulated spectral fit shown in Figure 1 was used, or whether the features were fit with one (or more?) Gaussian lines to derive the integrated intensities. The authors need to better explain their fitting process for these features.

3. The authors assume that interstellar abundance ratios match directly with those found in meteorites. While there are studies that show that ISM abundances may be relevant for protoplanetary disks, it is not clear this can be extended all the way to meteorites.

4. The authors invoke the late heavy bombardment period as a method for delivering organics to Earth. The timeline for impacts attributed to the late heavy bombardment period has recently come under scrutiny (see https://doi.org/10.1016/j.epsl.2022.117576). This should be included in the discussion.

Version 1:

Reviewer comments:

Reviewer #2

(Comments for the Author)
Abstract
The abstract describes this as the first detection of a “chiral, four-carbon ketose.” Notably, this would represent only the second chiral molecule detected in the ISM. However, beyond this mention, there is no further discussion of the significance of chirality—either in the context of molecular detections in space or its implications for prebiotic chemistry. Adding a brief discussion (a few sentences) on this point would strengthen the manuscript.

Figure 1
Figure 1 and the explanation in the text is much better and show the best, unblended transitions for this species. I still would only consider 4 transitions actually "clean" however - 42023, 40932, 33385 and 34639 MHz. With that being said, I would like the authors to describe in more detail how the 34639MHz line (and the other lines that say they are contaminated by a U-line) was determine to be blended by a U-line. Of the data presented in figure 1, that is one of the cleanest transitions detected. I have also read the methods section and the supplemental material and I don't follow how these transitions (and specifically the transition at 34639MHz) can be contaminated by a U-line.

Also in figure 1, the spectral line label are often buried inside the data being presented. For example, in panel d) you cannot even read the chemical formula for the transition around 38335MHz. If you insist on putting the chemical formulas or names that close to the spectrum, put them in a box so you can read what they are or move them around the panel so they are more visible.

Finally in Fig 1, panel f, you have a spectral feature of NH_2COCH_2OH labeled where there is nothing but a flat baseline. Similar to the analysis that you did for HC_9N, or C_7H, there is clearly no indication of this species in the spectrum so I would leave that out of the line fitting routine or explain why that feature is missing from that passband. Given the overall strength of the transition, it should definitely be there.

Discussion:
"… delivered to early Earth during the Late Heavy Bombardment"… this is interesting because you are suggesting that the molecules themselves would actually survive the impact intact. Is this possible give the size and fragility of the molecule to survive after undergoing those extreme conditions?

"…demonstrates that the ISM is a viable sugar feedstock"… I think this statement needs to be softened a bit given that, so far, this is the only ISM environment this molecule has been detected in - a very unique cloud near the Galactic center and is definitely not found in other regions of the Galaxy. The statement suggests it is a pretty ubiquitous species in the ISM which it clearly is not.

Methods:
There are several instances of … "by ref.^##"… I think this is a formatting error somewhere.

Rotation Diagram Analysis:
I appreciate the author's reply but in this case, it really does not add anything additional to the paper either in the analysis or identification. The MADCUBA analysis that was done was complete enough so I would remove this entire (4 sentence) section and figure.

Reviewer #3

(Comments for the Author)
The authors have submitted a revision of the manuscript that contains a much more extensive analysis and consideration of the statistical weight of the reported detection of erythrulose, a 4C sugar, in G+0.693. The first version of the manuscript did not clearly demonstrate a convincing detection of this sugar molecule in this source because many (or most) of the lines seemed to be blended with features from other molecules. However, the authors have now made a much more substantial case for this detection. They have reconsidered other potential molecules that could lead to line blending and removed several that were borderline detections. Once this was finished, they redid their analysis for erythrulose and find more "nearly unblended" lines to showcase in Figure 1. They also systematically check the results of their fitting against the standard in the field for confirming a detection. The sum total of this evidence now makes a case that this detection is valid. I appreciate the time and effort that went into this reevaluation and the careful nature with which they critiqued their own work. In my view they answered all of the concerns by all reviewers and present a stronger case for their findings. The manuscript in its current form now meets the standard for publication.

Open Access This Peer Review File is licensed under a Creative Commons Attribution 4.0 International License, which permits use, sharing, adaptation, distribution and reproduction in any medium or format, as long as you give appropriate credit to the original author(s) and the source, provide a link to the Creative Commons license, and indicate if changes were made.
In cases where reviewers are anonymous, credit should be given to 'Anonymous Referee' and the source.
The images or other third party material in this Peer Review File are included in the article's Creative Commons license, unless indicated otherwise in a credit line to the material. If material is not included in the article's Creative Commons license and your intended use is not permitted by statutory regulation or exceeds the permitted use, you will need to obtain permission directly from the copyright holder.
To view a copy of this license, visit https://creativecommons.org/licenses/by/4.0/

Reviewers' comments:

Reviewer #1 (Comments for the Author):

The features highlighted in Figure 1 are very compelling for the unique detection of this sugar, the first interstellar sugar. Hence, this journal is a fitting venue for this exciting paper. The supporting information from the quantum chemical computations on the surfaces as well as the kinetics modeling give a clear indication that this molecule should be able to be formed under the conditions of the observed region. Hence, the discovery has both firm and supportive evidence. The manuscript is well constructed, and there were only a few items that I thought the authors could or should address.

The last paragraph of the "Introduction" is actually results and should be moved down a section. This leaves a thin introduction, but one that should be able to stand on its own.

--- Response ---

We thank the referee for this suggestion. We respectfully prefer to keep this paragraph in the Introduction because it provides context for the results presented later in the manuscript. In addition, we have tried to follow the typical style of recently accepted manuscripts in Nature Astronomy (see for instance, McClure et al. 2023 or Smith et al. 2025), where the sentence starting from "Here, we report..." appears before the "Results" section.

We have also tried to solve the issue of a short introduction by providing additional context on the recent discovery of bio-essential sugars (ribose and glucose) in asteroid Bennu by Furukawa et al. (2026) and by stressing the difficulties inherent in the laboratory determination of the millimeter spectroscopic information of these sugars for their subsequent search in astronomical sources. We hope that, with these changes, the Introduction provides now a broader context. See 2nd paragraph of the Introduction (in blue text).

--- Referee ---

The authors really need to cite some papers by other groups. For instance, the ice grain work producing sugars led by Kaiser should also be cited (DOI: 10.1126/sciadv.adl323 & DOI: 10.1073/pnas.2320215121). Additionally, a couple of citations to recent detections by the group led by Cernicharo are warranted such as the detection of trans-propenethial.

--- Response ---

We thank the referee for the suggestion of the first article. We believe it is the work by Wang et al. (2024), with DOI:10.1126/sciadv.adl3236 (note that the final number

is missing in the referee report). As shown by these laboratory experiments, sugar acids can be synthesized experimentally by the energetic processing of interstellar ice analogues containing ethylene glycol and CO_2, through the formation of the radical e*. We have introduced a sentence about this in the 1st paragraph of Section "Formation mechanism on interstellar ice surfaces", see blue text in lines 133-135.

The second article from R. Kaiser's group by Zhang et al. (2024), was already cited in the Discussion section, but we now cite both papers in the Discussion section together. See blue text in Line 237 of the manuscript.

Regarding the works by Cernicharo's group, we now cite in the first sentence of the Introduction section the papers by Cernicharo et al. (2021), Wenzel et al. (2025) and Fuentetaja et al. (2026), referring to the largest aromatic species detected in the ISM by the group of J. Cernicharo and B. McGuire. See blue text in Line 31 of the manuscript.

--- Referee ---

Several reference calls go to superscripts that should be on the line of the text.

--- Response ---

We are sorry for this, but this is the editing style of Nature Astronomy when using the command "ref. \cite{citation}" in the Latex template.

--- Referee ---

The computational methods section has a discussion about the positions of the molecules. The text should point to Figure 2 to make this more concrete.

--- Response ---

Thank you for the suggestion. In the Computational Methods section, we now point to Figure 2 twice in lines 361 and 365 (see blue text).

---- Referee ----

Reviewer #2 (Comments for the Author):

Overall, the authors are making a bold new claim presenting evidence for the first detection of an actual sugar - Erythrulose - detected in the gas phase toward the high mass molecular cloud G+0.693.

The data the authors have collected on this source is remarkable - covering frequencies from about 35 - 150 GHz and the chemistry in this source is definitely unique as it contains molecular species in the gas phase that are not detected in any other astronomical environment including NH_2OH and unique S-bearing molecules such as HCOSH and CH_3SCH_3. The explanation for this unique

molecular complexity is attributed to wide-spread shocks pervasive in the region that leads to the sputtering of unique chemical species off grain surfaces so they can be detectable in the gas-phase.

The observing team over the course of many years has provided evidence for some of the most complex astronomical molecules ever detected - surpassing even the molecular complexity found in Sgr B2N. And in this work, they again present evidence for one of the largest organic astronomical molecules ever claimed which would have profound implications on understanding the nature and formation of sugars, and possibly even amino acids, under interstellar conditions. If shown to be correct, it would be one of the most significant contributions to the field of astrochemistry and deserve publication in Nature Astronomy. Unfortunately, after critical review of the data presented, there is just not enough evidence present to make such an revolutionary claim. I strongly recommend the authors continue on this path started in this work and gather additional evidence in support of this detection.

--- Response ---

We thank the referee for their careful and constructive review, which has helped us to strengthen the analysis and presentation of our results. In response, we provide additional and more rigorous evidence supporting the detection of erythrulose.

We now include all erythrulose transitions covered by our dataset. Figure 1 presents the brightest and least blended transitions, with integrated intensities exceeding 9σ, where σ corresponds to the uncertainty in the integrated intensity (defined as rms × √(delta_v × FWHM), with delta_v = 256 kHz and FWHM = 22 km s^{-1}). These transitions correspond to 12 spectral features, of which 6 (with 9 individual transitions of erythrulose) are classified as predominantly unblended, with contamination levels below 25%. This ensures that the emission in these features is dominated by erythrulose and provides a robust basis for identification. These 12 features are used to derive the updated LTE model presented in Table 1.

In addition, Extended Data Figure 1 shows the remaining 36 erythrulose transitions within the dataset, all with peak intensities above 1.2 mK (three times the average rms noise level in the spectra shown in Figure 1). Although these transitions are weaker or more affected by blending/noise and are therefore not included in the LTE fitting, all predicted features are consistently reproduced by the model. The agreement across the full set of detected transitions provides strong, independent validation of the identification.

We further emphasize that, although G+0.693 is chemically rich, its physical conditions differ significantly from those of hot cores and hot corinos. The molecular emission toward G+0.693 is sub-thermally excitated, particularly for

complex organic molecules with large dipole moments. This is due to the moderate gas densities of the cloud (of a few 10^4 cm^{-3}; e.g. Zeng et al. 2020). This substantially reduces line crowding and spectral confusion compared to warmer sources, where higher-energy and vibrationally excited transitions dominate.

Within this context, the detection of 6 largely unblended features, corresponding to 9 independent transitions of erythrulose, provides a high level of confidence (>99%) in its identification. Taken together, the inclusion of the full set of transitions, the consistency of all observed features with the LTE predictions, and the new quantitative treatment of line blending, strengthens the robustness of our result.

This is now better explained in the 1st, 2nd, 3rd and 5th paragraphs of the Results section (see blue text).

---- Referee ----

Major concerns:

The data presented in Figure 1 of the proposed 27 transitions do not show any clear detection of even 1 individual transition that can be defined as "clean" by either the criteria references by the authors themselves or from previous studies (Snyder et al. 2005 & Xue et al. 2019 - the accepted standard in the field for what can be considered an unblended transition). The first identification of an actual interstellar sugar - glycolaldehyde has often been referred to as a sugar but definitely is not by formal chemical definitions - would be remarkable. As such, a remarkable claim requires remarkable evidence. That remarkable evidence is not presented in these data. Especially when you compare the data presented in Fig 1 with the supplemental data presented on ethylene glycol where multiple, well defined and clean transitions are present in the data.

---- Response ----

Ethylene glycol's abundance is a factor of 3.5 more abundant than erythrulose and its dipole moments are also larger than those of erythrulose, which explains the bright lines observed for ethylene glycol with peak intensities larger than 30 mK. The two molecules are thus not expected to present equally-strong spectra.

We apologize for not explaining in detail the method used to identify the mostly unblended transitions of erythrulose.

In the previous version of the manuscript, we used the criteria presented in Rey-Montejo et al. (2024), which are based on the accepted standard of Snyder et al. (2005) and further expanded by Halfen et al. (2006). In this new version, we have revised these criteria and we now employ more stringent ones to those of Rey-Montejo et al. (2024). First, the transitions considered for the LTE fit of erythrulose are the brightest and least contaminated features with integrated intensities >9σ

(see above). In addition, to calculate the residuals (and hence, the level of line blending), we now consider the velocity range of +-FWHM (or over 2xFWHM, which covers 98.2% of the total area of the Gaussian line), while in Rey-Montejo et al. (2024) we used a velocity range of +-FWHM/2 (or over a FWHM). As shown below in the response to referee #3, in the most unfavourable case with a flat spectrum, the residuals are expected to be of about 50%. Therefore, we chose a threshold of residuals of less than half this value (<25%) to classify those transitions as mostly unblended.

We explain in detail the criteria used to select the most unblended transitions of erythrulose in the new Section "Identification of Unblended Transitions" in Methods (see blue text), which also considers other factors: i) accurate rest frequencies, ii) frequency agreement of the observed features, iii) beam dilution, iv) linewidth agreement, v) relative intensities, vi) confirmation of transitions, and vii) level of blending.

Following these criteria, we now identify a cleaner subset of transitions, which are the ones shown in Figure 1 and in Extended Data Table 1, which correspond to the brightest and most unblended features that have been used to derive the new LTE fit of erythrulose. In this new subset, 6 out of them are classified as mostly unblended features, accounting for 9 individual transitions of erythrulose. In addition, another 3 features are blended with U-line emission and the last 3 appear blended with known molecules. However, Figure 1 shows that the new LTE fit reproduces well all the observed features and it provides consistent predictions for the observed spectra of Extended Data Figure 1. Note that even blended features with residual emission >=25%, such as those shown in panels b, d and h in Figure 1 (i.e. the sets of transitions 8_{7,1}->7_{6,1}+8_{7,2}->7_{6,2}, and 7_{6,1}->6_{5,1}+7_{6,2}->6_{5,2}, and for the 13_{2,12}->12_{2,11} line), are well reproduced by the global LTE fit. All this supports the robust identification of erythrulose.

The 12 sets of transitions selected to obtain the LTE fit of erythrulose are now shown in Figure 1 and their derived line parameters are listed in Extended Data Table 1. The description about how we have performed the LTE fit of erythrulose is described in detail in Section "Detection of erythrulose in G+0.693" (see blue text).

---- Referee ----

In addition, there are several molecules that are labeled in the spectrum as possible interloper species - looking at just the top row of figure 1, there are supposed interlopers of CH3C6H, HCCCH2CN, HC9N and c-C6H5CN. These species have largely been found in cold dark clouds and while the conditions presented in G+0.693 may be similar to a dark cloud environment, such large hydrocarbons are not typically found in regions as massive and complex as G+0.693.

---- Response ----

We would like to thank the referee for identifying this potential issue. HCCCH2CN is detected in G+0.693 as reported in Rivilla et al. (2022, Front. Astron. Space Sci. 9, 876870). However, we have revised the LTE fits of CH3C6H, HC9N, C7H and c-C6H5CN.

CH3C6H had an inaccurate upper limit and, after a proper calculation, it does not contribute any longer to the observed feature at 42023 MHz. Thanks to this change, the brightest erythrulose feature at 42023 MHz appears dominated by the contribution of erythrulose, with only a small contribution from U-line emission of <25% (see new Figure 1).

For HC9N, there are some hints of detection but its robust assignment cannot be confirmed with the current dataset. Therefore, we consider this molecule as a non-detection in our analysis. As a result, the contribution of HC9N to the observed feature at 35443 MHz is negligible and, thanks to the new LTE fit of erythrulose, this feature is found to be mostly unblended (contribution by U-line emission of 20%; see Extended Data Table 1 and Figure 1).

We have also revised the upper limit of C7H, which appeared in the panel with the transition at 32356 MHz in the previous version of Figure 1. With the new upper limit, this species does not contribute any longer and hence, the feature observed at this frequency corresponds solely to erythrulose (see panel q in new Extended Data Figure 1).

Although not published yet, c-C6H5CN is clearly detected in our G+0.693 dataset. We include below a figure with the detected lines of c-C6H5CN, together with the LTE fit predicted by MADCUBA-SLIM. c-C6H5CN presents several lines that are clean and the LTE fit reproduces well the observed features. The work presenting the detection of c-C6H5CN is currently in preparation (Rivilla et al.). Note that we have recently reported the discovery of the first sulfur-bearing cyclic molecule in the ISM (Araki et al. 2026), indicating that G+0.693 presents an active carbon-chain and aromatic chemistry similar to that found in cold molecular clouds such as TMC-1.

After revising the upper limits of CH3C6H, HC9N, and C7H, we have re-fitted the erythrulose line emission yielding Tex = 11.3+-1.8 K and N(erythrulose) = (8.7+-0.8) x 10^{13} cm^{-2}, which are similar to the values we had before.

We have generated a new Figure 1 and the new physical parameters of erythrulose have been updated in Table 1 of the manuscript.

[redacted]

---- Referee ----

Conversely, there is NO evidence for the transition of CH3COOH around 45.010 GHz - which I would assume to be present because there is both a label and modeled spectral feature… yet the spectrum at that frequency is complete blank showing no evidence of acetic acid.

---- Response ----

The blue line profile shown in that panel at 45.010 GHz corresponds to the LTE emission predicted by MADCUBA for the physical properties derived for CH3COOH and reported in Sanz-Novo et al. (2023, ApJ, 954, 3). That blue line profile, while being consistent with the observed spectrum, lies below the rms noise level for that particular transition. To avoid confusion, we have slightly increased the threshold used to display the labels of the detected species. As a result, the CH3COOH label at 45.010 GHz is no longer shown in the updated version (see new panel i in Extended Data Figure 1). We hope this improves the clarity of the manuscript.

---- Referee ----

Overall, the proposed identification a new sugar species is called into question given the lack of ANY clear, clean and unblended transitions as well as the questionable identification of transitions of other molecular species that are 1) clearly not represented at all in the spectrum presented and 2) would be the first identification of a species in a region that is not a cold, dark cloud (or in this region with considerably less uv-flux and where shocks are pervasive.).

---- Response ----

As presented above, we now report a total of 12 sets of erythrulose lines that are well fitted by our LTE model, and with 6 out of which are mostly unblended (with a contamination of <25%).

To strengthen the identification of erythrulose, we also include the calculation of the probability of chance alignment of the detected features. Assuming the confusion limit and Gaussian line profiles, the chance of finding a feature at the VLSR of G+0.693 with a tolerance of +-4 km s-1 (i.e. the largest velocity shift observed for the molecular line emission in G+0.693; Jimenez-Serra et al. 2022; Colzi et al. 2022) is p = 36% for a velocity coverage equivalent to the FWHM of the erythrulose lines. The detection of each successive line has a probability p^n with n the number of unblended transitions. Considering six mostly unblended features, the probability of chance alignment is p^6=0.2%, which implies a confidence level of 1-p^6=99.8% for the detection of erythrulose. Even if three or four unblended lines were considered, we would still obtain confidence levels of 95.2% and 98.3%, respectively. Note that this estimate is conservative since line confusion has not been reached yet in our G+0.693 spectral surveys, which provides additional support to the identification of erythrulose in this cloud. Also, note that, although G+0.693 presents line-rich and complex spectra, the molecular emission in this cloud is sub-thermally excited with low Tex, which yields much lower levels of line blending and line confusion as compared to hotter sources such as massive hot cores and low-mass hot corinos.

This discussion has been included in the 5th paragraph of Section "Detection of erythrulose in G+0.693".

Finally, we would like to mention that despite the high-levels of extinction in this cloud, the cosmic-ray ionization rate in the Galactic Center is a factor >100 higher (of 1.3x10^-15 s-1) than in the Galactic disk (standard value of 1.3x10^-17 s-1), which generates a secondary UV-photon radiation field a factor of >100 stronger in G+0693 than that found in typical Galactic disk molecular clouds such as TMC-1.

---- Referee ----

It is good that the authors present the spectroscopic data in the supplemental material but the full spectroscopic catalog would also be necessary to include so

that a full analysis can be done by the reader. The authors also make the claim that no other transitions are missing from any of the other data in their survey as they either are severely blended and/or lie below the measured noise level. Presenting even some of these additional transitions/data to the reader would provide a better case for the data presented in figure 1 as "clean" transitions.

---- Response ----

We now present the remaining transitions of erythrulose covered within our dataset with peak intensities >1.2 mK, i.e. 3 times the average rms noise level measured in the Yebes 40m data (see new Extended Data Figure 1). These lines are weaker or more affected by blending and/or noise and were hence not used in the LTE fit of erythrulose. As can be seen from Extended Data Figure 1, the LTE fit of this C4 sugar is consistent with the observed spectra for all covered transitions.

---- Referee ----

My assumption, without actually seeing any of these additional data, is that the blended transitions throughout the 2 and 3mm bands would not look any worse than the data presented in Figure 1 - which again is fueling my skepticism that there are any clear transitions of this molecule present in these data - and even if there was 1 completely clean transition, would that be enough to make the claim of the detection of a new sugar molecule? ...

--- Response ---

The transitions present in the Q-band at 7mm are expected to be the brightest for the low Tex derived for this large molecule. A similar behaviour has been seen for other large COMs also reported toward this cloud (see e.g. Araki et al. 2026).

In any case, we now present all transitions covered in our dataset, including the 3mm lines expected to be the brightest according to the LTE fit of erythrulose. We have marked these transitions in Extended Data Figure 1 with blue asterisks. As seen from this Figure, the 3mm transitions appear heavily blended, but the LTE fit nicely matches the observed spectra (see for instance, the set of transitions 14_{11,3}->13_{10,3}+14_{11,4}->13_{10,4} in panel s of Extended Data Figure 1).

We stress these points in the 3rd paragraph of the Results section (see lines 86-91).

--- Referee ---

... That is doubtful as a region that is as complex as G+0.693 would contain hundreds (if not thousands) of U-lines that may be attributed to other well-known, yet uncharacterized species (e.g. isotopologues, higher vibrational states). Finally, it should be stressed again that the presentation of a rotation diagram provides not

additional evidence for a claimed new detection and should not be used in support of a new detection.

---- Response ----

Over 180 species, including isotopologues, are considered in our LTE model within MADCUBA. This is highlighted in blue in the 1st paragraph of the Results section:

"...while in blue we report the total spectrum predicted by MADCUBA-SLIM considering the contribution from all molecular species identified and modelled toward G+0.693 (more than 180 species, including isotopologues, are considered in the model)"

The results of the isotopologue emission will be published elsewhere (Colzi et al., in prep).

Regarding the higher vibrational states, it is important to note that the Tex of the emission of erythrulose, and of other COMs, in G+0.693 is <15K, which makes impossible the presence of transitions from vibrationally-excited states. This is one of the reasons why G+0.693 represents an excellent laboratory to search for new species since its spectrum suffers much less from line blending and line confusion compared to hotter sources such as hot molecular cores or hot corinos.

Given that we have re-analysed the detected features of erythrulose, we have updated the rotation diagram shown in Extended Data Figure 2, where we use the brightest and mostly unblended transitions employed to derive the LTE fit with MADCUBA-SLIM and shown in Figure 1 and Extended Data Table 1. The rotation diagram method (Goldsmith & Langer 1999) is used as an alternative analysis method to derive the physical parameters of the erythrulose emission, because this is the method traditionally employed in other works (see e.g. Belloche et al. 2013, 2019). It is re-assuring that the detected erythrulose features yield a rotation diagram whose derived physical parameters are similar to those obtained by using MADCUBA-SLIM (the derived parameters are Tex=10.5+-1.8 K and N=(9.3+-2.9)x10^{13} cm^{-2}.

---- Referee ----

Finally, the modeling work done looks complete and extensive. The authors have done an amazing amount of work trying to fundamentally explain the possible formation of a 4-C sugar in this environment. However, it is beyond my area of expertise to comment on the actual chemical formation routes to the proposed formation routes to 3- and 4-carbon sugars. What I can comment on is the calculated relative abundance ratios between the claimed detected molecules with respect to the model predictions. If I am reading figure 3 correctly, the abundance of Erythrulose is not orders of magnitude larger than the 3-C sugars... at any time or

in any model. Yet, the observed abundances are (at least) 2 orders of magnitude different. The authors make several assumptions as to why the 3- and 4-C sugars may be different in abundance given things like the 3-C sugars are more readily destroyed in the gas-phase and that both species may be harder to get off the grain surface or rapidly re-absorb onto the surface.

The issue however is clear, there is no fundamental reason given, from a chemical kinetics perspective, as to why this particular 4-C sugar should be preferentially formed with respect to other chemical similar (and simpler) species. Which again, really calls into question as to whether the species is there at all in the data at the claimed abundance. Given the formation models present in this work, the 3-C sugars should absolutely be present under these conditions - may be a factor of 2 lower in abundance at the most but the lack of any evidence for the simpler, smaller species without any good evidence or explanation, again, calls into question the detection of one of the largest, and most complex sugar ever claimed.

---- Response ----

As acknowledged in the first version of the manuscript, the chemical modelling is subject to large uncertainties given the lack of knowledge of important parameters such as the UV-photodissociation efficiencies of C3 and C4 sugars, reaction branching ratios or even missing reactions. In addition, it is important to note that the KMC simulations do not include gas-phase chemistry and hence, C3 and C4 sugars may undergo efficient gas-phase destruction through ion-neutral reactions after the ice is released in the G+0.693 shock. This idea is supported by the recent work of Lema-Saavedra et al. (2025), where glyceraldehyde is found to be more unstable than glycolaldehyde in the gas phase, which may explain the lack of detection of the former species.

We have stressed this point in the 4th paragraph of Section “Astrochemical modelling of erythrulose formation”:

“In addition, note that the KMC simulations do not include an extended gas-phase chemical network for these COMs and hence, C3 and C4 sugars may undergo efficient gas-phase destruction through ion-neutral reactions (Sanz-Novo et al. 2024) after the ice release in the shock. Glyceraldehyde has recently been found to be more unstable than glycolaldehyde in the gas phase, which may explain its lack of detection (Lema-Saavedra et al. 2025).”

Although our chemical modelling does not match exactly the observed gas-phase abundances, note that the predicted abundances of CH3OH, ethylene glycol glycolaldehyde and erythrulose in our KMC simulations lie within a factor of 5 with respect to the observed ones, well within the tolerance of one order of magnitude

typically assumed for chemical modelling. This is now emphasized in the 3rd paragraph of Section "Astrochemical modelling of erythrulose formation":

"In our simulations, the predicted abundances in the ice of CH3OH, glycolaldehyde, ethylene glycol, and erythrulose, all lie within a factor of 5 of the observed values (Table 1) and well within the tolerance of one order of magnitude typically considered in chemical modelling"

In addition, we have added the values of the modelled abundances of these molecules, together with those of the C3 sugars, in a new column in Table 1 (see blue text). Although the predicted abundances of the C3 sugars are factors 25-70 different from the observed ones, the abundance ratios of glycolaldehyde, ethylene glycol and of the C3 sugars with respect to erythrulose differ by, at most, a bit more than one order of magnitude, being fairly consistent given the large uncertainties of our simulations.

See also the new Table 2, where we now show the abundance ratios of glycolaldehyde, ethylene glycol and of the C3 sugars with respect to erythrulose for both the observations and the model (abundances obtained at 1.6 My from the model with zeta = 1.3x10^-14 s-1).

---- Referee ----

Reviewer #3 (Comments for the Author):

The manuscript NATASTRON-25091222, "Sugar in interstellar space" by Jimenez-Serra et al. reports the interstellar detection of erythrulose, a four-carbon ketose sugar, in the molecular cloud G+0.693-0.027. No other sugar has yet been detected in the ISM, despite many sugars being found in the carbonaceous material in meteorites. This result is of great potential interest in the fields of astrochemistry and astrobiology, as sugars are essential biomolecules and their formation on early Earth is poorly understood. The detection of this particular sugar is also surprising, as searches for smaller three-carbon sugars glyceraldehyde and dihydroxyacetone in this and other sources have not previously led to detections.

The authors showcase their results with a fair and balanced presentation of the difficulties in detecting such a molecule in a dense spectrum containing many lines from numerous interstellar species. They then go through a detailed analysis of possible formation mechanisms to explain the unexpectedly high fractional abundance of this molecule relative to other related species previously detected in this source.

Despite many positive aspects of the manuscript, I have serious reservations about the interpretation of the observational results. Examining Figure 1 in detail reveals that most of the brightest erythrulose lines that are reported herein are actually

significantly blended with lines arising from other known molecules. The lines that are not blended are considerably weaker than the others. And yet the authors claim that there are unblended lines of significant intensity -- where? I do not see them in Figure 1. Confused by the author's claim that "[f]ourteen out of 27 transitions are either clean or slightly blended with other molecular species" I turned to Extended Data Table 1. And this is where I found the source of my confusion. Most of the features listed here as "clean" are actually blended features in the observational spectra. The distinction is that they are blended with features that have not yet been identified. An example is the feature at 40.93 GHz, which is listed in this table as clean and shown in Figure 1 as the third panel from the left in the top row. While indeed this feature is clean from other *known* lines, the modeled spectrum in red only accounts for about half of the integrated intensity in the observed feature. The rest of the intensity must be arising from a different molecule that has not yet been identified.

There are many such features shown in Figure 1. Simply put, these are not clean lines -- these are blended lines. Labeling them as clean leads to confusion at best and misinterpretation of the the results at worst. This calls into question the validity of the detection. And that calls into question the necessity for the rest of the study. If this detection is not valid, then the molecular abundance derived is not reliable, and the rest of the analysis and interpretation is based on incomplete or erroneous information. The authors need to better justify their claim of a detection before the rest of the manuscript can be considered.

---- Response ----

As described above, we have carried out a re-analysis of the data, where some interlopers with inaccurate upper limits (in particular, CH3C6H, HC9N, and C7H) do not contribute any longer to the features assigned to erythrulose. This has allowed us to obtain a better LTE fit of these features. We now use more stringent criteria to the one employed in Rey-Montejo et al. (2024), which followed the criteria of Snyder et al. (2005) and Halfen et al. (2006) (the standards in the field). This includes a new calculation of the residuals considering a velocity range of 2xFWHM, and adopt a convention by which the features with a contamination <25% are classified as mostly unblended.

To illustrate the selection of this threshold, we have performed numerical simulations of mock spectra with flat line profiles (representing the most extreme case of line blending) and with slightly-broader Gaussian line profiles with a linewidth = 1.3 x the original linewidth and a peak intensity = 1.1 x the original peak intensity (representing a slightly-blended mock spectrum). This is shown in the Figure below:

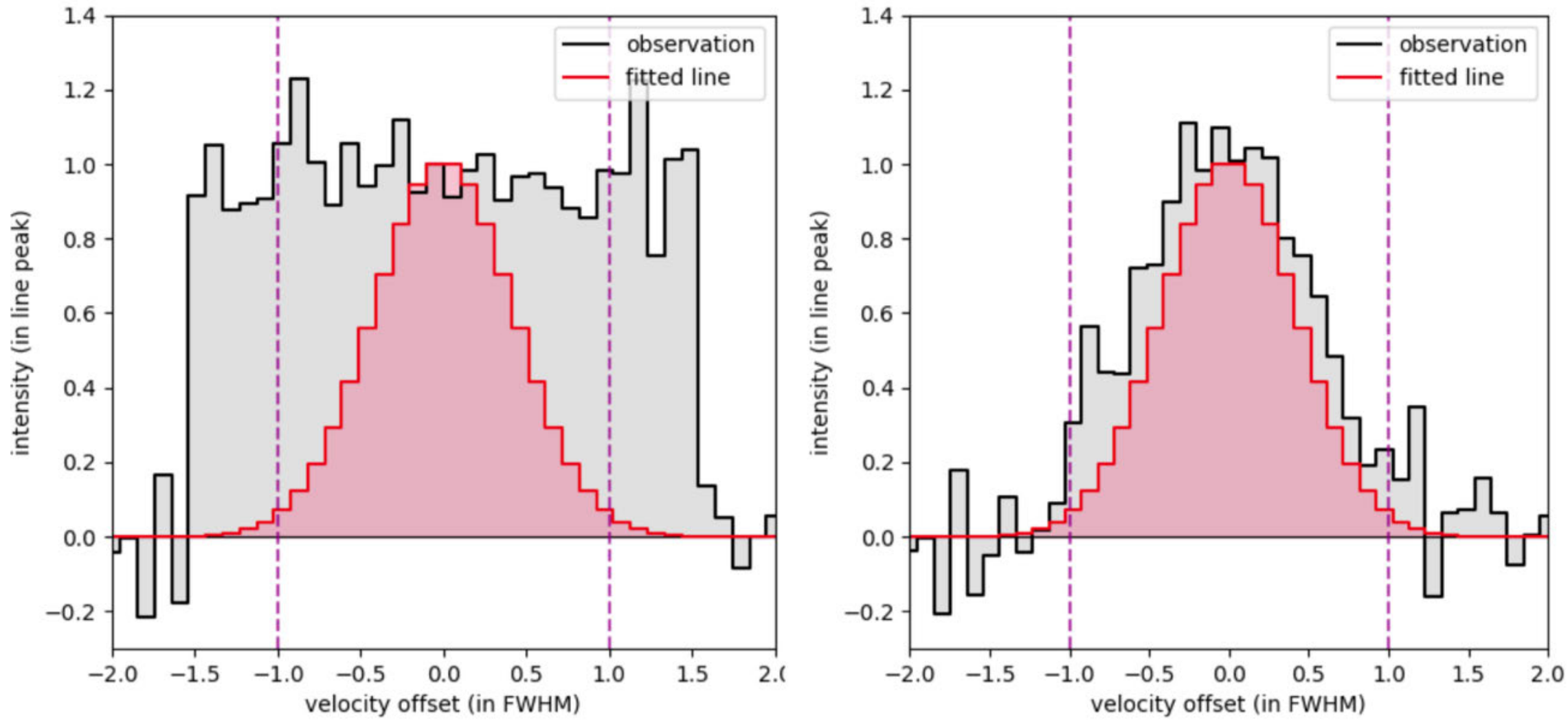

The residuals obtained by subtracting the area of the original Gaussian line (red histogram and shaded area) across a velocity range of +-FWHM (or over 2 x FWHM) give 50% of contamination for the flat mock spectrum. However, for the slightly-blended case, the residuals go down to less than 25%, providing a robust justification for our choice of threshold for establishing the features classified as mostly-unblended.

This is now explained in detail in the new section "Threshold to establish the level of line blending" in the Supplementary Material (see blue text).

Following this procedure, we have identified a total of 6 mostly-unblended features that account for 9 individual transitions of erythrulose. We hope it is now clearer that the major contributor to the features shown in Figure 1 is erythrulose.

We also calculate the confidence level of the detection of erythrulose using the six mostly-unblended features and obtain 99.8%, which provides strong statistical support for the identification of erythrulose. The probability of chance alignment of these 6 features is indeed very low (0.2%) even in the conservative case of line confusion, which has not been reached in the spectra of G+0.693.

---- Referee ----

If one proceeds through the rest of the manuscript based on the assumption that the detection is valid, the other steps of the analysis and interpretation are well done. Additional comments that could further enhance the rest of the manuscript include:

1. The statement "Given that glyceraldehyde and dihydroxyacetone cannot serve as precursor molecules for erythrulose, we turn our attention to smaller building blocks of this C4 sugar." needs to be backed up with more information and references. Why can these two smaller sugars not serve as precursors to erythrulose?

---- Response ----

It is unlikely that glyceraldehyde and dihydroxyacetone are parent molecules of erythrulose because the upper limits to their abundance are factors of >8-17 lower than that of erythrulose. We have tried to explain this better at the beginning of Section "Formation mechanism on interstellar ice surfaces". See the new 1st sentence in blue in this Section.

"The C3 sugars glyceraldehyde and dihydroxyacetone are factors ≥8-17 less abundant than erythrulose, which suggests that they are unlikely to be its dominant precursors under the physical conditions of G+0.693. Therefore, we turn our attention to smaller and more abundant building blocks of this C4 sugar such as glycolaldehyde and ethylene glycol."

---- Referee ----

2. In the rotation diagram analysis presented on page 7, the authors did not make clear whether the simulated spectral fit shown in Figure 1 was used, or whether the features were fit with one (or more?) Gaussian lines to derive the integrated intensities. The authors need to better explain their fitting process for these features.

---- Response ----

In "Methods", we now explain that the rotation diagram was calculated using only the brightest and most-unblended transitions shown in Figure 1 and in Extended Data Table 1, considering the velocity-integrated intensity over the linewidth (Rivilla et al. 2021). See blue text in new Lines 351-353.

---- Referee ----

3. The authors assume that interstellar abundance ratios match directly with those found in meteorites. While there are studies that show that ISM abundances may be relevant for protoplanetary disks, it is not clear this can be extended all the way to meteorites.

---- Response ----

The results from the Rosetta mission have shown that the organic inventories of comets are similar to those measured in presolar environments, indicating a connection between the chemistry observed in the ISM and that inherited by minor bodies of the Solar System (see e.g. Altwegg et al. 2019, ARA&A, 57, 113). A good example of the link between the ISM and meteorites is ethanolamine, another important prebiotic molecule reported also for the first time toward G+0.693 (Rivilla et al. 2021, PNAS, 118, 22). As presented by these authors, the meteoritic

ethanolamine/H_2O abundance ratio is 3×10^{-6}, very similar to that derived toward G+0.693 (of 1×10^{-6}). We explain this better in the “Discussion” section:

Lines 230-232: “The similarity of the organic inventories of comets and presolar environments indicates a connection between the chemistry observed in the ISM and that inherited by minor bodies of the Solar System (Altwegg et al. 2019).”

Lines 246-248: “Other prebiotic organics such as ethanolamine, also detected in G+0.693, have been found in meteorites with abundance ratios with respect to water similar to those measured in the ISM (Rivilla et al. 2021).”

---- Referee ----

4. The authors invoke the late heavy bombardment period as a method for delivering organics to Earth. The timeline for impacts attributed to the late heavy bombardment period has recently come under scrutiny (see https://doi.org/10.1016/j.epsl.2022.117576). This should be included in the discussion.

---- Response ----

We thank the referee for the suggestion. We now add this information in Lines 251-254 of the main text:

“Although the extent and intensity for the Late Heavy Bombardment period has recently been questioned (Cartwright et al. 2022), recent Monte-Carlo simulations of Earth’s impact history show that biopoiesis could occur within a much broader timeline between 4.45 and 3.9 billion years ago with >16% chance (Wogan et al. 2024)”

Dear Editor,

We would like to thank the reviewers for their positive reports and for their additional input. We have addressed all their remaining comments as described below.

Yours sincerely,
Izaskun Jiménez-Serra

Reviewer #2:
Comments for the Author:
Abstract
The abstract describes this as the first detection of a "chiral, four-carbon ketose". Notably, this would represent only the second chiral molecule detected in the ISM. However, beyond this mention, there is no further discussion of the significance of chirality - either in the context of molecular detections in space or its implications for prebiotic chemistry. Adding a brief discussion (a few sentences) on this point would strengthen the manuscript.

---- Response ----

We thank the reviewer for this suggestion. We have added a couple of sentences in the Discussion section stressing the importance and implications of detecting a second chiral molecule in the ISM. We have also included in Section 2.2 two comments regarding the chirality nature of erythrulose for the study of its formation mechanism on interstellar ices. See text in blue in Section 2.2 and in the Discussion section.

---- Reviewer ----

Figure 1
Figure 1 and the explanation in the text is much better and show the best, unblended transitions for this species. I still would only consider 4 transitions actually "clean" however - 42023, 40932, 33385 and 34639 MHz. With that being said, I would like the authors to describe in more detail how the 34639MHz line (and the other lines that say they are contaminated by a U-line) was determine to be blended by a U-line. Of the data presented in figure 1, that is one of the cleanest transitions detected. I have also read the methods section and the supplemental material and I don't follow how these transitions (and specifically the transition at 34639MHz) can be contaminated by a U-line.

---- Response ----

In point vii) of Section "Identification of Predominantly Unblended Transitions" in Methods, we now explain in more detail how we calculate the residuals after subtracting the LTE fit area of each erythrulose line to the observed spectrum and explicitly indicate that we adopt a criteria by which an observed feature is considered as mostly unblended if the residual area lies below 25%. This threshold is justified by our mock simulations shown in Supplementary Information and Supplementary Figure 2. For the transition at 34639MHz, the residuals contribute with 35% to the total area of the observed feature and therefore, following the criteria described above, this line is considered as blended with U-line emission.

---- Reviewer ----

Also in figure 1, the spectral line label are often buried inside the data being presented. For example, in panel d) you cannot even read the chemical formula for the transition around 38335MHz. If you insist on putting the chemical formulas or names that close to the spectrum, put them in a box so you can read what they are or move them around the panel so they are more visible.

---- Response ----

We thank the referee for pointing out this issue. We have shifted the labels in the panels so that they can be read properly.

---- Reviewer ----

Finally in Fig 1, panel f, you have a spectral feature of NH2COCH2OH labeled where there is nothing but a flat baseline. Similar to the analysis that you did for HC9N, or C7H, there is clearly no indication of this species in the spectrum so I would leave that out of the line fitting routine or explain why that feature is missing from that passband. Given the overall strength of the transition, it should definitely be there.

---- Response ----

Thank you for spotting this issue. This predicted feature was produced mainly by CH3COCH3 with a minimal contribution from NH2COCH2OH (which was presented by Rivilla et al. 2023, ApJ, 953L, 20R). CH3COCH3, despite being clearly detected in our dataset, it has not been reported yet toward G+0.693 in any publication. The LTE fit of CH3COCH3 has been improved and, as can be seen from new Figure 1, panel f, the predicted intensity of this feature is much weaker and consistent with the noise of the observed spectrum.

---- Reviewer ----

Discussion:
"….delivered to early Earth during the Late Heavy Bombardment"… this is interesting because you are suggesting that the molecules themselves would actually survive the impact intact. Is this possible give the size and fragility of the molecule to survive after undergoing those extreme conditions?

---- Response ----

Laboratory experiments simulating meteorite impact carried out by McCaffrey et al. (2014, Orig Life Evol Biosph, 44, 29) and by Zellner et al. (2020, Astrobiology, 20, 11), show that 95% of the initial glycolaldehyde included in the experiments can survive impact delivery for pressures between 4.5 and 25 GPa. This was already mentioned in the previous version of the manuscript together with the reference by McCaffrey et al. (2014). We now include an additional reference with the more recent experiments by Zellner et al. (2020), which show that small sugars and sugar derivatives can also survive meteoritic impact. See new text in blue in the Discussion section.

---- Reviewer ----

"…demonstrates that the ISM is a viable sugar feedstock"… I think this statement needs to be softened a bit given that, so far, this is the only ISM environment this molecule has been detected in - a very unique cloud near the Galactic center and is definitely not found in other regions of the Galaxy. The statement suggests it is a pretty ubiquitous species in the ISM which it clearly is not.

---- Response ----

We have tried to soften this statement by changing the verb from its "is" form to the "could be" form. In addition, we have softened the last sentence of the abstract which now states that "interstellar erythrulose could have contributed to the sugar inventory available for early metabolic and replication processes."

---- Reviewer ----

Methods:
There are several instances of … "by ref.^##"… I think this is a formatting error somewhere.

---- Response ----

We have fixed this formatting error and we believe the new version of the manuscript does not present this problem.

---- Reviewer ----

Rotation Diagram Analysis:
I appreciate the author's reply but in this case, it really does not add anything additional to the paper either in the analysis or identification. The MADCUBA analysis that was done was complete enough so I would remove this entire (4 sentence) section and figure.

---- Response ----

Following the referee's suggestion, we have removed the section explaining the rotation diagram method and the previous Extended Data Figure 2 reporting the rotation diagram of erythrulose. For consistency, we have also removed the rotation diagram of ethylene glycol (previous Supplementary Figure 2). Any reference to the rotation diagrams in the article has also been removed.

---- Reviewer ----

Reviewer #3:
Comments for the Author:
The authors have submitted a revision of the manuscript that contains a much more extensive analysis and consideration of the statistical weight of the reported detection of erythrulose, a 4C sugar, in G+0.693. The first version of the manuscript did not clearly demonstrate a convincing detection of this sugar molecule in this source because many (or most) of the lines seemed to be blended with features from other molecules. However, the authors have now made a much more substantial case for this detection. They have reconsidered other potential molecules that could lead to line blending and removed several that were borderline detections. Once this was finished, they redid their analysis for erythrulose and find more "nearly unblended" lines to showcase in

Figure 1. They also systematically check the results of their fitting against the standard in the field for confirming a detection. The sum total of this evidence now makes a case that this detection is valid. I appreciate the time and effort that went into this reevaluation and the careful nature with which they critiqued their own work. In my view they answered all of the concerns by all reviewers and present a stronger case for their findings. The manuscript in its current form now meets the standard for publication.

---- Response ----

We thank the reviewer for their positive answer and for providing an initial report that helped to improve the manuscript.

---- Additional comment from the authors ----

We would like to mention that we have modified the value of the VLSR of erythulose in Table 1 to 69 km s-1, which was fixed in the LTE fit performed by MADCUBA in the previous version of the manuscript. This was indicated in the main text (Line 94) and in Methods (see point ii) in Section "Identification of Predominantly Unblended Transitions"), but we forgot to update this value in Table 1 in the previous version. This is why we are changing it now. We apologize for the inconvenience.
